# Antennal transcriptome analysis of the piercing moth *Oraesia emarginata* (Lepidoptera: Noctuidae)

**DOI:** 10.1371/journal.pone.0179433

**Published:** 2017-06-14

**Authors:** Bo Feng, Qianshuang Guo, Kaidi Zheng, Yuanxia Qin, Yongjun Du

**Affiliations:** 1 Institute of Health and Environmental Ecology, Wenzhou Medical University, University Town, Wenzhou, Zhejiang, China; 2 Department of Research and Development, Newcon Inc., Ningbo, Zhejiang, China; USDA Agricultural Research Service, UNITED STATES

## Abstract

The piercing fruit moth *Oraesia emarginata* is an economically significant pest; however, our understanding of its olfactory mechanisms in infestation is limited. The present study conducted antennal transcriptome analysis of olfactory genes using real-time quantitative reverse transcription PCR analysis (RT-qPCR). We identified a total of 104 candidate chemosensory genes from several gene families, including 35 olfactory receptors (ORs), 41 odorant-binding proteins, 20 chemosensory proteins, 6 ionotropic receptors, and 2 sensory neuron membrane proteins. Seven candidate pheromone receptors (PRs) and 3 candidate pheromone-binding proteins (PBPs) for sex pheromone recognition were found. *OemaOR29* and *OemaPBP1* had the highest fragments per kb per million fragments (FPKM) values in all ORs and OBPs, respectively. Eighteen olfactory genes were upregulated in females, including 5 candidate PRs, and 20 olfactory genes were upregulated in males, including 2 candidate PRs (*OemaOR29* and *4*) and 2 PBPs (*OemaPBP1* and *3*). These genes may have roles in mediating sex-specific behaviors. Most candidate olfactory genes of sex pheromone recognition (except *OemaOR29* and *OemaPBP3*) in *O*. *emarginata* were not clustered with those of studied noctuid species (type I pheromone). In addition, *OemaOR29* was belonged to cluster PRIII, which comprise proteins that recognize type II pheromones instead of type I pheromones. The structure and function of olfactory genes that encode sex pheromones in *O*. *emarginata* might thus differ from those of other studied noctuids. The findings of the present study may help explain the molecular mechanism underlying olfaction and the evolution of olfactory genes encoding sex pheromones in *O*. *emarginata*.

## Introduction

Olfaction plays a key role in foraging [1–3], mating [4,5], and oviposition behaviors [6–8] of insects. Insect olfaction studies have provided fundamental insights into chemosensory biology and chemical ecology and have provided valuable opportunities for pest management [9–14]. Lepidopterans are often used for olfaction studies, as these have extensive and sensitive olfactory repertoires. However, molecular studies on olfaction in Lepidopterans lag behind those of other insect models such as fruit fly and mosquitos [15].

Lepidoptera sex pheromones are divided into two main types based on their chemistry [16]. Type I pheromone components have 10- to 18-carbon, even numbered straight chain acetates, aldehydes, and alcohols. Type II pheromones consist of polyunsaturated C_17_-C_23_ straight chains, skipped conjugated polyenic hydrocarbons and the corresponding epoxide derivatives [17]. Type I pheromones occur in about 75% of all studied moth species, whereas type II pheromones occur in about 15% of identified Lepidopteran pheromones [17]. These two major types of sex pheromones are produced through distinct pathways that involve different biosynthetic sites, substrates, and enzymes, as well as respectively employ specific endocrine regulatory mechanisms. However, both types of pheromones have the same function in mate recognition and attraction in moths [16,18].

Genes encoding Lepidopteran olfactory proteins have been identified in *Bombyx mori* [19], and also in the pest species *Manduca sexta* [20], *Heliothis virescens* [21], *Spodoptera litura* [22], *S*. *littoralis* [23,24], *Agrotis ipsilon* [25], and *Dendrolimus spp*. [26]. Sex pheromones of above species are type I. However, studies on the olfactory genes that encode type II pheromones are limited.

**Fig 1:**
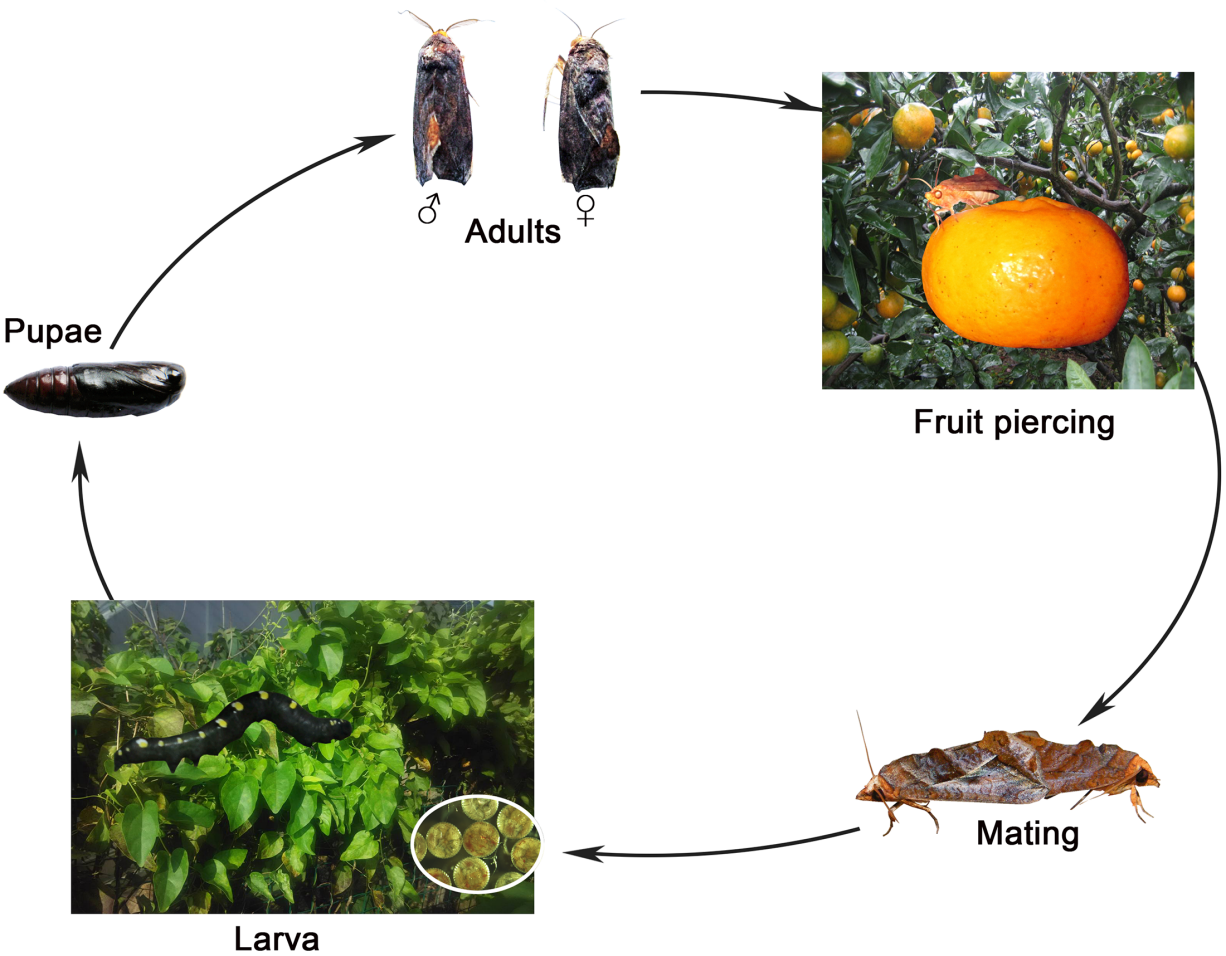
Life cycle of *O*. *emarginata*.

The piercing fruit moth *Oraesia emarginata* Fabricius (Lepidoptera: Noctuidae) is an important pest of fruits such as citrus, pear, peach, and plum. The larvae feed on plants belonging to the Menispermaceae. Adult moths obtain nutrition from ripe fruits. Mated females lay eggs on Menispermaceae plants (Fig 1) [27]. The electroantennographic and behavioral responses of *O*. *emarginata* to volatiles from ripe fruits [28] and the repellency of a volatile compound, sec-butyl β-styryl ketone have been studied [29]. However, little is known about the olfactory mechanism of *O*. *emarginata*. Type II pheromones were identified as female sex pheromones in *Oraesia* species. The major and minor sex pheromone components of the related *O*. *excavate* were identified as cis-9,10-epoxy-(Z)-6 –heneicosene and cis-9,10-epoxy-(Z,Z)-3,6- heneicosadiene [30]. Although the sex pheromone of female *O*. *emarginata* was not published, it was similar to epoxide components from a preliminary identification (Du et al., unpublished data). In the present study, we achieved significant coverage of olfactory genes with *de novo* transcriptome and measured gene expression using real-time quantitative reverse transcription PCR analysis (RT-qPCR) for comparison between the sexes. We also discuss the diversification of olfactory genes for the recognition of type I and type II pheromones.

## Materials and methods

### Insects

*O*. *emarginata* larvae were collected from fields in Gannan City of Jiangxi Province, China and reared in the laboratory at 25 ± 1°C and 75 ± 5% relative humidity with a 14-h light/10-h dark photoperiod. Our field collection activities did not impact endangered or protected species. Larvae were fed fresh leaves of *Cocculus orbiculatus* until pupation. Emergence of males and females was checked every morning, and adults were separately maintained in ventilated wooden cages (35 cm × 35 cm × 50 cm). Emerging adult moths were fed with 10% glucose water soaked into cotton.

### Extraction of total RNA from tissues

Antennae of 4-d-old adults were used. A total of 25 adults (males and females separately) were collected after 3.5 h of the dark cycle. Antennae samples from each group were immediately homogenized in TRNzol-A+ (TIANGEN Biotech, Beijing, China) on ice, and total RNA was extracted according to the manufacturer’s instructions. The concentration and purity of the total RNA were determined by using a NanoDrop2000 spectrophotometer (ThermoFisher, Waltham, MA, USA). RNA with an A260/A280 ratio between 1.75–2.05, an A260/A230 ratio > 1, and a concentration > 400 ng/μL was used for the experiments. Total RNA was treated with DNase I (Takara, Kusatsu, Shiga, Japan) to remove any genomic DNA. RNA extractions were performed in triplicate.

### *De novo* transcriptome analysis

The same amount of RNA collected from male and female antennae was pooled for transcriptome analysis. The cDNA library for transcriptome analysis was prepared using a TruSeq SBS Kit v3-HS (Illumina, San Diego, CA, USA), following the manufacturer’s recommendations. The library was sequenced using Illumina HiSeq^™^ 2000 (Illumina, San Diego, CA, USA) with a 90-bp read length for the paired-end reads by BGI (Shenzhen, Guangdong, China). Dirty reads containing adapters and unknown or low-quality bases were discarded from the raw reads to obtain clean reads for analysis. *De novo* transcriptome assembly was conducted with the short reads assembly program, Trinity (r20140413p1, min_kmer_cov:2) [31]. BLASTx (v2.2.28+) alignment (E value < 0.00001) between unigenes and protein databases (NCBI non-redundant protein database, Swiss-Prot, Kyoto Encyclopedia of Genes and Genome (KEGG), and Clusters of Orthologous Groups (COG)) was successively performed. Gene ontology (GO) annotations of the unigenes were determined using Blast2go (http://www.blast2go.org/) [32].

### Olfactory gene analysis

The candidate olfactory gene was manually obtained from gene annotation. In addition, a 50% ORF length cutoff was used in identifying putative genes to prevent a gene from being counted twice. The candidate OBPs and CSPs were searched for the presence of N-terminal signal peptides using SignalP4.0 (http://www.cbs.dtu.dk/services/SignalP/) using default parameters [33]. The signal peptides likely contained significant phylogenetic information and were included in the phylogenetic analyses of OBPs and CSPs [34]. Amino acid sequence alignment was performed using CLUSTALX2.1 using default parameters [35]. For phylogenetic analysis, known amino acid sequences of olfactory genes from other insects were downloaded (S1 File). Phylogenetic analyses were conducted using the maximum likelihood method of MEGA 6.0, which was based on the Jones-Taylor-Thornton (JTT) substitution model, partial deletion gaps with 95% site coverage cutoff, a nearest neighbor interchanges (NNI) heuristic search, and other default parameters [36]. Node support for the phylogenetic tree was assessed using the bootstrap method with 1,000 bootstrap replicates.

### Profiling analysis of gene expression based on the antennal transcriptome

Gene expression levels were calculated using the fragments per kb per million fragments (FPKM) method based on the results of antennal transcriptome analysis. The number of fragments that uniquely aligned to a gene was divided by the total number of fragments that uniquely aligned to all genes and by the base number in the CDS of that gene [37]. The FPKM method can eliminate the influence of different gene lengths and sequencing levels on the calculation of gene expression.

### RT-qPCR analysis of olfactory gene expression in the antennae

Single-stranded cDNAs were synthesized from 1 μg of total RNA using the ReverTra Ace qPCR RT Kit (Toyobo, Kita-ku, Osaka, Japan) following the manufacturer’s recommendations. RT-qPCR was performed with SsoFast^™^ EvaGreen^®^ Supermix (Bio-Rad, Hercules, CA, USA), following the manufacturer’s protocols, in a CFX-96^™^ PCR Detection System (Bio-Rad). The cycling conditions were an initial cycle at 95°C for 30 s, followed by 39 cycles of 95°C for 5 s and 60°C for 5 s. Dissociation curves with 0.3°C/s melt rates were used to check for the presence of non-specific dsDNA SYBR Green hybrids. Only primers with a single PCR amplification product were used in the subsequent analyses. The amplification efficiency of each primer was calculated from the slope of the standard curve [38]. The PCR primers used are listed in S1 Table. Ubiquinol-cytochrome c reductase (*UCCR*) and arginine kinase (*AK*) were used as reference genes. The difference in gene expression was measured by using the 2^-ΔΔCt^ algorithm [39]. Differential gene expression between females and males was measured, with the female antennae used as reference. Expression levels of target genes were normalized independent of each reference gene with the algorithm, and then averaged. When the gene expression of the female antennae was very low, the gene expression of the male antennae was used as control. RNA extraction was repeated three times for each sample, and two or more RT-qPCR replicates were prepared for each sample.

### Data analysis

Data analysis was conducted using SPSS 17.0 (SPSS Inc., Chicago, IL, USA). The significance of the difference between means was determined using the student’s *t*-test. The critical P value for each test was set at 0.05.

## Results

### *De novo* antennal transcriptome assembly

Using the Illumina HiSeq^™^ 2000 sequencing system, 117,410,034 raw reads were obtained from the antennal samples. After removing low-quality (< Q20) adaptor and contaminating sequence reads, 103,301,292 (a total of 9,297,116,280 bp) clean reads were generated from antennae, and 42,992 unigenes were assembled (N50 = 1,098), with a mean length of 713 bp. More than 58% (24,954) of the unigenes were aligned to sequences in various protein databases. GO annotation was performed to obtain information on their molecular function, biological process, and cellular location (S1 Fig). The raw sequence of the transcriptome has been deposited to the National Center for Biotechnology Information (NCBI) (GenBank Accession Number PRJNA358570; https://www.ncbi.nlm.nih.gov/bioproject/PRJNA358570).

### Analysis of olfactory genes

**Fig 2:**
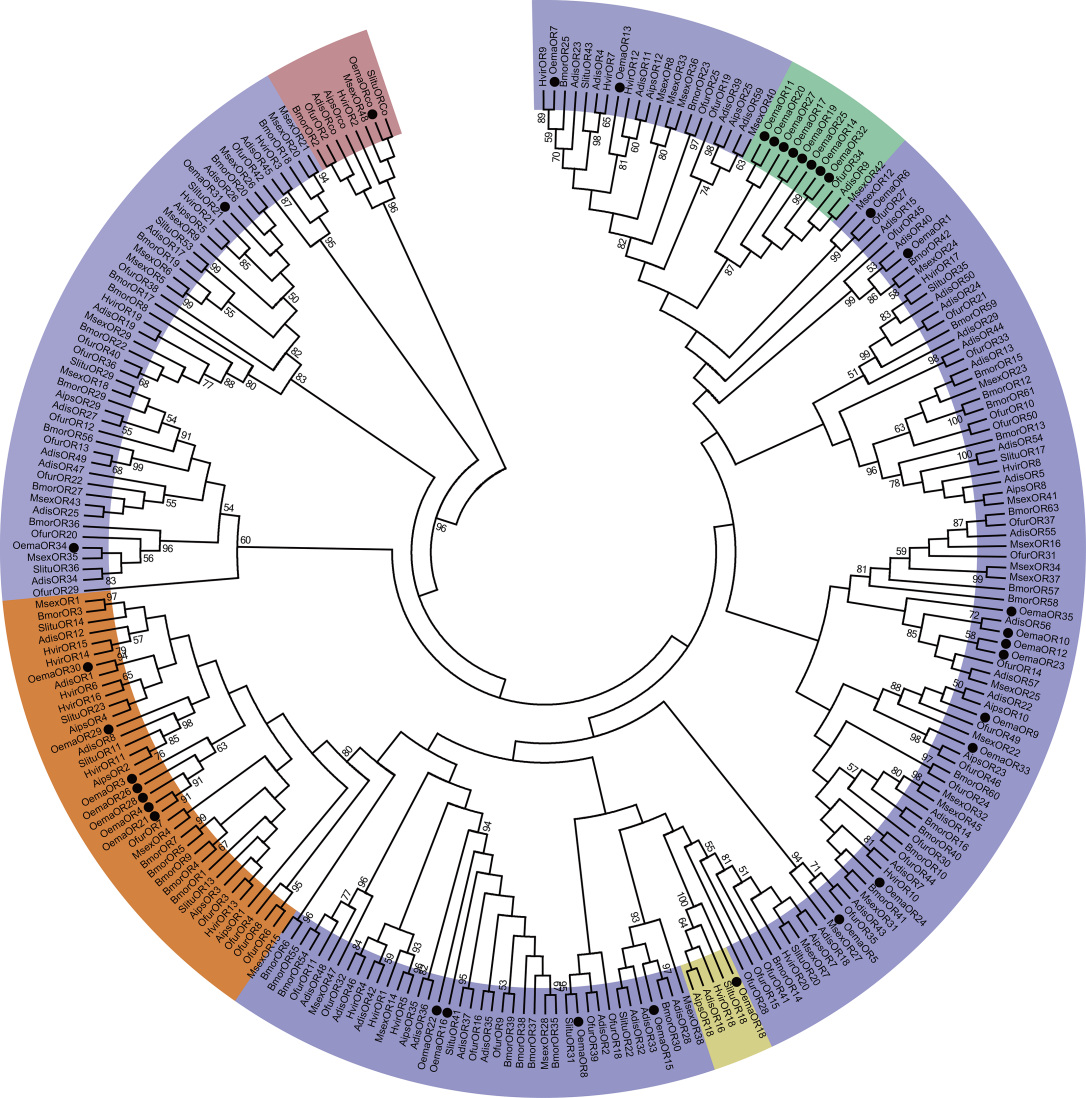
Phylogenetic analysis of putative OR gene sequences of *O*. *emarginata* (black circle). The tree was rooted with Orco lineage (pink color). Bootstrap values < 50% are not shown. Color legend: Orange = PR group, yellow = OR18 group, green = OemaORs group, and blue = other general OR groups. Adis, *Athetis dissimilis*, Aips, *A*. *ipsilon*, Bmor, *B*. *mori*, Hvir, *H*. *virescens*, Msex, *M*. *sexta*, Oema, *O*. *emarginata*, Ofur, *O*. *furnacalis*, Slitu, *S*. *litura*.

The 35 candidate OR genes encoding an olfactory receptor co-receptor (*OemaORco*), *OemaOR18*, 7 candidate pheromone receptors (PRs, *OemaOR3*, *4*, *21*, *26*, *28*, *29*, and *30*) and 26 general OR genes were identified from *O*. *emarginata* antennae (Table 1, Fig 2). Candidate PRs of *O*. *emarginata* were clustered together with previously reported PRs in the phylogenetic tree. Eight general ORs (*OemaOR11*, *14*, *17*, *19*, *20*, *25*, *27*, and *32*) were clustered with *OfurOR34*, *MsexOR42*, and *AdisOR9* into a specific group, with a bootstrap support value of 87 (Fig 2). Two general OR genes (*OemaOR24* and *35*) were not clustered with any reported ORs from Lepidopteran species with sufficient bootstrap values (bootstrap values <50). Full open reading frame (ORF) of 8 OR genes (*OemaOR5*, *9*, *19*, *22*, *26*, *29*, *35* and *ORco*) were obtained, with the mean length of 435 aa.

**Table 1:** BLASTp results of candidate olfactory receptors of *O*. *emarginata*.

Gene name	Full ORF	Group	FPKM	Gene length (aa)	Reference gene ID	Reference gene name	E_value	Similarity (%)
*OemaOR1*	No	General	6.1	271	AII01102.1	Odorant receptor [*Dendrolimus kikuchii*]	4.54E-129	70.1
*OemaOR3*	No	Pheromone	10.0	269	AGS41448.1	Olfactory receptor 9 [*A*. *segetum*]	2.25E-32	24.9
*OemaOR4*	No	Pheromone	7.0	299	AGY14585.2	Putative odorant receptor [*Sesamia inferens*]	2.98E-81	45.5
*OemaOR5*	Yes	General	6.6	402	AGG08877.1	Putative olfactory receptor 44 [*S*. *litur*a]	0	83.8
*OemaOR6*	Yes	General	6.7	392	BAR43469.1	Putative olfactory receptor 27 [*Ostrinia furnacalis*]	0	78.1
*OemaOR7*	No	General	9.6	329	CAD31950.1	Putative chemosensory receptor 9 [*H*. *virescens*]	4.02E-95	47.4
*OemaOR8*	No	General	3.6	207	AIG51892.1	Odorant receptor [*Helicoverpa armigera*]	3.38E-121	82.6
*OemaOR9*	Yes	General	13.9	437	AIG51891.1	Odorant receptor, partial [*H*. *armigera*]	0	65.9
*OemaOR10*	No	General	4.1	249	AIG51890.1	Odorant receptor [*H*. *armigera*]	6.71E-117	63.5
*OemaOR11*	No	General	7.5	194	AJD81541.1	Olfactory receptor 1, partial [*H*. *assulta*]	4.75E-77	56.7
*OemaOR12*	No	General	13.5	277	AII01072.1	Odorant receptor [*D*. *houi*]	4.55E-130	65.0
*OemaOR13*	No	General	9.9	358	AGK90004.1	Olfactory receptor 12 [*H*. *armigera*]	1.70E-137	53.2
*OemaOR14*	No	General	13.2	274	AGG08878.1	Putative olfactory receptor 12 [*S*. *litura*]	3.28E-115	62.8
*OemaOR15*	No	General	1.7	289	AIG51902.1	Odorant receptor, partial [*H*. *armigera*]	2.38E-108	54.7
*OemaOR16*	No	General	9.2	251	AIG51898.1	Odorant receptor [*H*. *armiger*a]	1.19E-75	49.8
*OemaOR17*	No	General	9.0	369	ABQ84982.1	Chemosensory receptor 12 [*S*. *littoralis*]	3.46E-129	50.1
*OemaOR18*	No	General	10.6	353	ACL81186.1	Putative olfactory receptor 18 [*H*. *ze*a]	1.17E-175	69.4
*OemaOR19*	Yes	General	3.5	463	AGG08878.1	Putative olfactory receptor 12 [*S*. *litura*]	3.47E-148	45.4
*OemaOR20*	No	General	5.6	248	ABQ84982.1	Chemosensory receptor 12 [*S*. *littoralis*]	1.23E-72	47.6
*OemaOR21*	No	Pheromone	4.5	266	AGI96751.1	Olfactory receptor 16 [*S*. *litura*]	9.95E-80	46.2
*OemaOR22*	Yes	General	10.9	424	AFL70813.1	Odorant receptor 50, partial [*M*. *sexta*]	1.05E-123	44.6
*OemaOR23*	No	General	5.9	237	AII01083.1	Odorant receptor [*D*. *kikuchii*]	7.66E-99	59.9
*OemaOR24*	No	General	6.7	308	AIG51858.1	Odorant receptor, partial [*H*. *armigera*]	3.39E-90	43.5
*OemaOR25*	No	General	17.1	339	ABQ84982.1	Chemosensory receptor 12 [*S*. *littoralis*]	1.49E-131	62.6
*OemaOR26*	Yes	Pheromone	8.4	447	AGK90019.1	Olfactory receptor 14b [*H*. *assulta*]	2.51E-131	46.3
*OemaOR27*	No	General	19.1	392	AGG08878.1	Putative olfactory receptor 12 [*S*. *litura*]	5.13E-142	50.8
*OemaOR28*	No	Pheromone	6.5	276	ACL81180.1	Putative olfactory receptor 11 [*S*. *littoralis*]	5.16E-54	37.3
*OemaOR29*	Yes	Pheromone	39.1	467	AGH58120.1	Odorant receptor 11 [*S*. *exigua*]	1.04E-180	53.5
*OemaOR30*	No	General	6.7	259	AIG51856.1	Odorant receptor [*H*. *armigera*]	7.40E-49	32.8
*OemaOR31*	No	General	4.5	197	AIG51896.1	Odorant receptor, partial [*H*. *armigera*]	3.70E-39	36.5
*OemaOR32*	No	General	15.1	390	AGG08878.1	Putative olfactory receptor 12 [*S*. *litura*]	1.72E-129	47.4
*OemaOR33*	No	General	6.0	223	BAR43488.1	Putative olfactory receptor 46 [*O*. *furnacalis*]	2.22E-73	61.9
*OemaOR34*	No	General	8.0	259	BAR43462.1	Putative olfactory receptor 20 [*O*. *furnacalis*]	4.32E-121	73.7
*OemaOR35*	Yes	General	15.3	413	KOB71190	Olfactory receptor 29 [*Operophtera brumata*]	0.00E+00	78.0
*OemaORco*	Yes	ORco	51.5	476	AFI25169.1	Odorant receptor 83b [*H*. *viriplaca*]	0.00E+00	93.5

**Fig 3:**
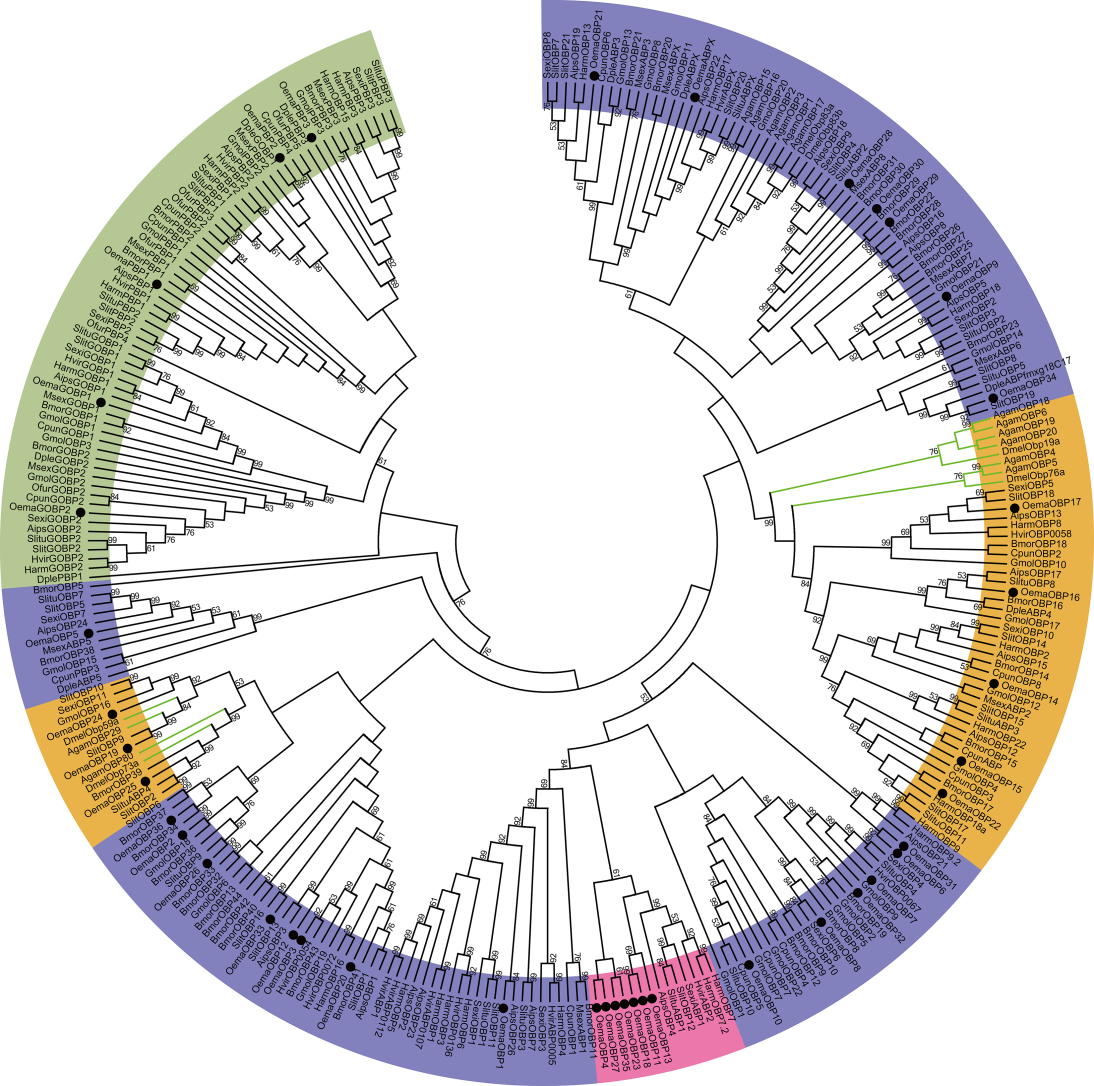
Phylogenetic analysis of putative OBP gene sequences of *O*. *emarginata* (black circle), other moth species (black lines), and Dipteran species (green lines). The tree was rooted with the Lepidopteran GOBP-PBP group (green color). Bootstrap values < 50% are not shown. Color legend: Orange = conserved OBP groups, pink = expanded OemaOBPs group, green = Lepidopteran GOBP-PBP group, and blue = other general OBP groups. Adis, *A*. *dissimilis*, Agam, *Anopheles gambiae*, Aips, *A*. *ipsilon*, Bmor, *B*. *mori*, Cpun, *Conogethes punctiferalis*, Dmel, *Drosophila melanogaster*, Dple, *D*. *plexippus*, Gmol, *Grapholita molesta*, Harm, *H*. *armigera*, Hvir, *H*. *virescens*, Msex, *M*. *sexta*, Ofur, *O*. *furnacalis*, Oema, *O*. *emarginata*, Sexi, *S*. *exigua*, Slit, *S*. *littoralis*, Slitu, *S*. *litura*.

**Fig 4:**
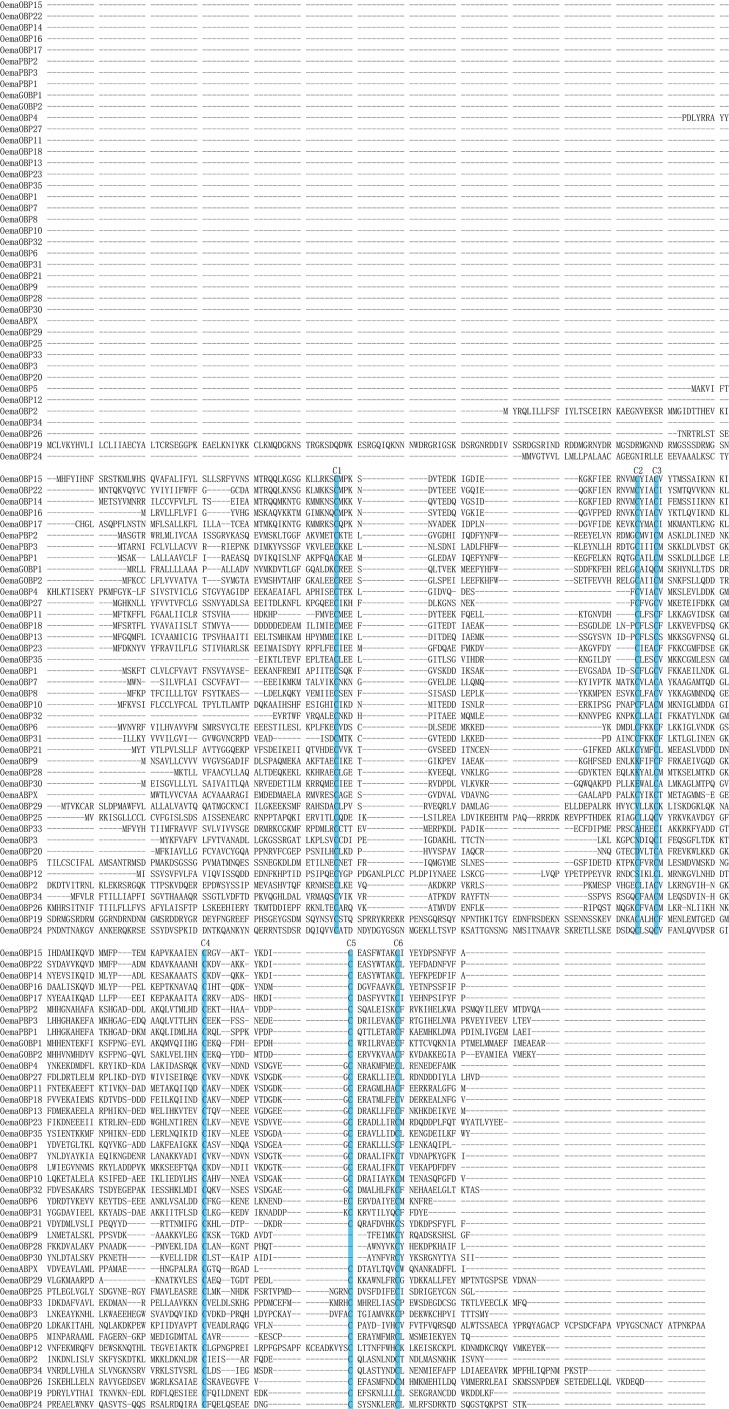
Aligned putative full ORF of OBP gene sequences of *O*. *emarginata*. Six conserved cysteines are highlighted in blue.

The 41 candidate odorant-binding protein (OBP) genes were identified from *O*. *emarginata* antennae. and these encoded 34 OBPs, 2 general odorant-binding proteins (GOBPs), 3 pheromone-binding proteins (PBPs), an antennal-binding protein (*OemaABPX*), and *OemaOBP25* (*DmelOBP73a* analogue) (Table 2, Fig 3). All OemaOBPs were clustered with those of Lepidopteran species with sufficient bootstrap values (bootstrap values > 60). Seven OemaOBP genes (*OemaOBP4*, *11*, *13*, *18*, *23*, *27*, and *35*) were clustered with *AipsOBP4*, *SlitABP1*, *SlitOBP12*, *SexiABP1*, *HvirABP2*, *HarmOBP7*, and *HarmOBP7*.*2* with a bootstrap support value of 61, and the latter 7 OBPs were clustered into a subgroup with a bootstrap support value of 99 (Fig 3). The mean length of the OBPs was 166 aa, and the full ORF of the 37 OBP genes were obtained. Thirty-three OBPs were a classic group with six conserved cysteines, 3 OBPs (*OemaOBP9*, *28*, and *30*) were of the minus-C group with C2 and C5 missing, and 5 OBPs (*OemaOBP3*, 12, 20, 29 and *33*) were of the plus-C OBP group with more than six conserved cysteines (Fig 4).

**Table 2:** BLASTp results of candidate odorant-binding proteins of *O*. *emarginata*.

Gene name	Full ORF	Group	FPKM	ORF length (aa)	Reference gene ID	Reference gene name	E_value	Similarity (%)
*OemaOBP1*	Yes	Classic	2833	148	AEB54581	OBP5 [*H*. *armigera*]	1.78E-58	64.2
*OemaOBP2*	Yes	Classic	24	210	EHJ64212	Odorant-binding protein 2 [*Danaus plexippus*]	3.99E-80	72.9
*OemaOBP3*	Yes	Plus	33	155	AGK24580	Odorant-binding protein 4 [*Chilo suppressalis*]	2.82E-65	60.6
*OemaOBP4*	Yes	Classic	7	161	AEB54591	OBP7 [*H*. *armigera*]	3.09E-17	33.5
*OemaOBP5*	Yes	Classic	1436	178	AGS36751	OBP10, partial [*S*. *inferens*]	2.31E-57	49.4
*OemaOBP6*	Yes	Classic	196	142	AGC92789	Odorant-binding protein 9 [*H*. *assulta*]	1.45E-19	28.9
*OemaOBP7*	Yes	Classic	16	145	ADY17886	Odorant binding protein [*S*. *exigua*]	2.98E-69	67.6
*OemaOBP8*	Yes	Classic	11	147	AFM77984	Odorant binding protein 6 [*S*. *exigua*]	8.21E-53	61.9
*OemaOBP9*	Yes	Minus	113	146	AAL60425	Antennal binding protein 7 [*M*. *sexta*]	3.45E-44	56.8
*OemaOBP10*	Yes	Classic	1796	153	AGP03457	SexiOBP11 [*S*. *exigua*]	7.60E-79	71.9
*OemaOBP11*	Yes	Classic	28	139	AEB54591	OBP7 [*H*. *armigera*]	7.76E-22	38.8
*OemaOBP12*	Yes	Plus	60	200	AGC92793	Odorant-binding protein 19 [*H*. *assulta*]	1.04E-30	36.0
*OemaOBP13*	Yes	Classic	917	149	CAC33574	Antennal binding protein [*H*. *virescens*]	1.33E-29	37.3
*OemaOBP14*	Yes	Classic	312	147	AEB54586	OBP2 [*H*. *armigera*]	6.72E-72	69.4
*OemaOBP15*	Yes	Classic	119	146	AII00997	Odorant binding protein [*D*. *kikuchii*]	2.51E-66	62.3
*OemaOBP16*	Yes	Classic	1497	155	AGP03456	SexiOBP10 [*S*. *exigua*]	1.35E-64	68.6
*OemaOBP17*	Yes	Classic	1796	153	AFG73000	Odorant-binding protein 2 [*Cnaphalocrocis medinalis*]	4.76E-78	76.5
*OemaOBP18*	Yes	Classic	11	149	CAC33574	Antennal binding protein [*H*. *virescens*]	5.11E-31	40.3
*OemaOBP19*	Yes	Classic	15	334	XP_011559551	General odorant-binding protein 71-like [*Plutella xylostella*]	2.06E-80	73.7
*OemaOBP20*	Yes	Plus	37	189	AGR39564	Odorant binding protein 1, partial [*A*. *ipsilon*]	2.49E-55	46.6
*OemaOBP21*	Yes	Classic	9327	153	AGH70104	Odorant binding protein 8 [*S*. *exigua*]	1.32E-77	83.7
*OemaOBP22*	Yes	Classic	161	146	AAL60415	Antennal binding protein 4 [*M*. *sexta*]	1.50E-72	78.1
*OemaOBP23*	Yes	Classic	11	158	CAC33574	Antennal binding protein [*H*. *virescens*]	1.94E-14	36.1
*OemaOBP24*	Yes	Classic	81	248	AII00994	Odorant binding protein [*D*. *kikuchii*]	7.81E-88	59.0
*OemaOBP25*	Yes	Classic	3	184	AII00978	Odorant binding protein [*D*. *houi*]	2.22E-124	96.7
*OemaOBP26*	No	Classic	4	208	NP_001140186	Odorant-binding protein 2 precursor [*B*. *mori*]	1.04E-101	67.8
*OemaOBP27*	Yes	Classic	9	146	AEX07271	Odorant-binding protein [*H*. *assulta*]	2.25E-11	35.9
*OemaOBP28*	Yes	Minus	551	133	AGH70105	Odorant binding protein 9 [*S*. *exigua*]	8.22E-83	91.7
*OemaOBP29*	Yes	Plus	19	157	AGK24578	Odorant-binding protein 2 [*C*. *suppressalis*]	1.75E-16	74.4
*OemaOBP30*	Yes	Minus	4	141	AGK24581	Odorant-binding protein 5 [*C*. *suppressalis*]	2.49E-24	38.3
*OemaOBP31*	No	Classic	96	130	AGC92789	Odorant-binding protein 9 [*H*. *assulta*]	4.65E-09	26.2
*OemaOBP32*	No	Classic	4	127	AII00969	Odorant binding protein [*D*. *houi*]	6.62E-38	46.5
*OemaOBP33*	Yes	Plus	323	172	NP_001159621	Odorant binding protein LOC100307012 [*B*. *mori*]	4.88E-07	38.8
*OemaOBP34*	Yes	Classic	4	182	EHJ74351	Odorant-binding protein 2 [*D*. *plexippus*]	2.06E-102	79.7
*OemaOBP35*	No	Classic	5	123	AEX07270	Odorant-binding protein [*H*. *assulta*]	9.52E-16	34.1
*OemaABPX*	Yes	Classic	890	136	AGS36754	OBPABPX, partial [*S*. *inferens*]	2.62E-62	69.1
*GOemaOBP1*	Yes	Classic	1796	164	AAW65076	General odorant binding protein 1 [*H*. *assulta*]	1.16E-89	75.0
*GOemaOBP2*	Yes	Classic	1796	161	AIS72932	General odorant-binding protein 2 [*S*. *litura*]	4.06E-99	87.6
*OemaPBP1*	Yes	Classic	10342	166	AAC36315	Pheromone binding protein [*H*. *zea*]	6.90E-76	66.0
*OemaPBP2*	Yes	Classic	1796	168	AAF16710	Pheromone binding protein 2 [*M*. *sexta*]	5.17E-79	63.1
*OemaPBP3*	Yes	Classic	2245	163	AFM36758	Pheromone-binding protein 3 [*A*. *ipsilon*]	3.97E-78	66.3

**Fig 5:**
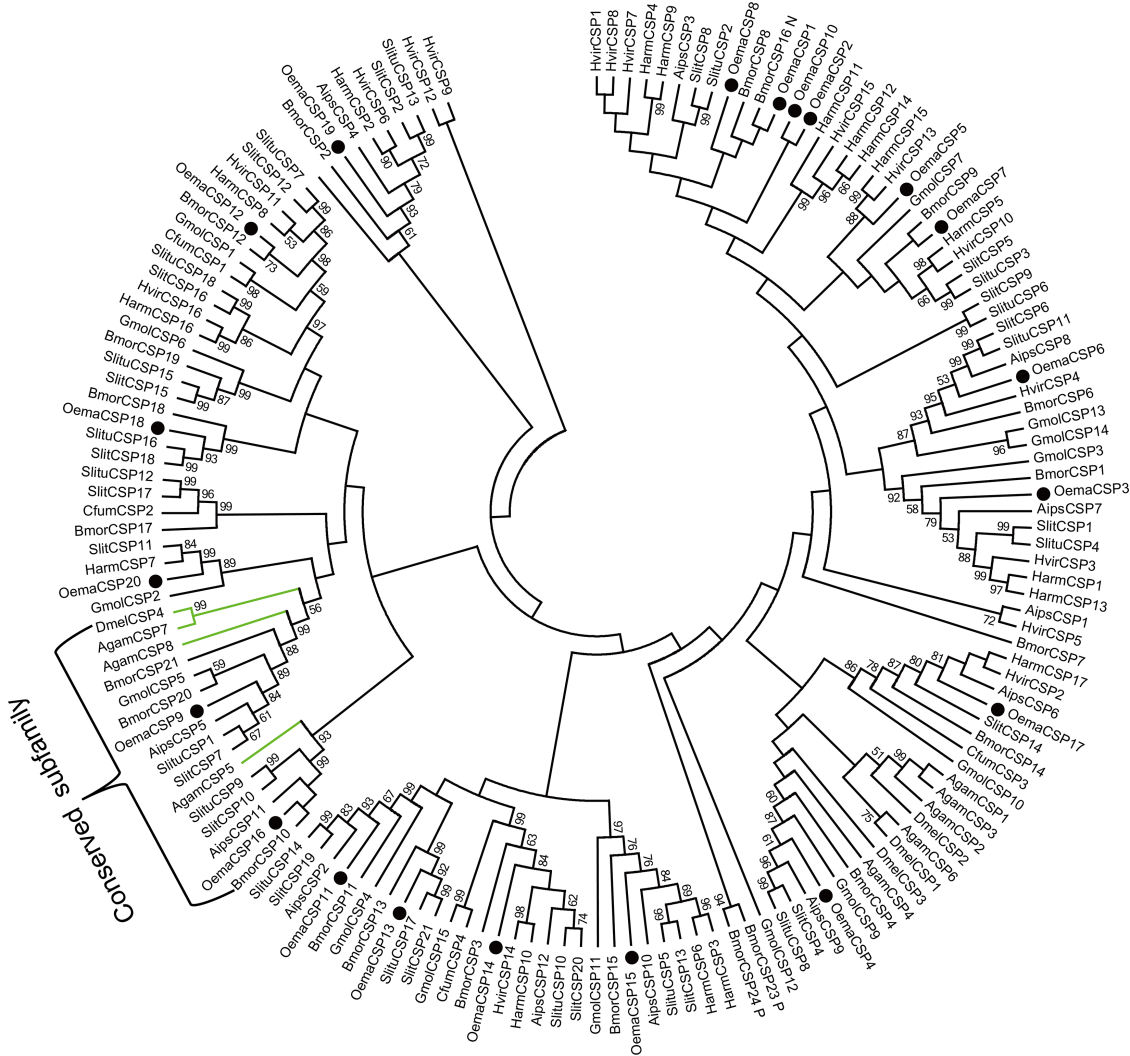
Phylogenetic analysis of putative CSP gene sequences of *O*. *emarginata* (black circles), other moth species (black lines) and Dipteran species (green lines). Bootstrap values < 50% are not shown. Agam, *A*. *gambiae*, Aips, *A*. *ipsilon*, Bmor, *B*. *mori*, Dmel, *D*. *melanogaster*, Gmol, *G*. *molesta*, Oema, *O*. *emarginata*, Slit, *S*. *littoralis*, Slitu, *S*. *litura*.

**Fig 6:**
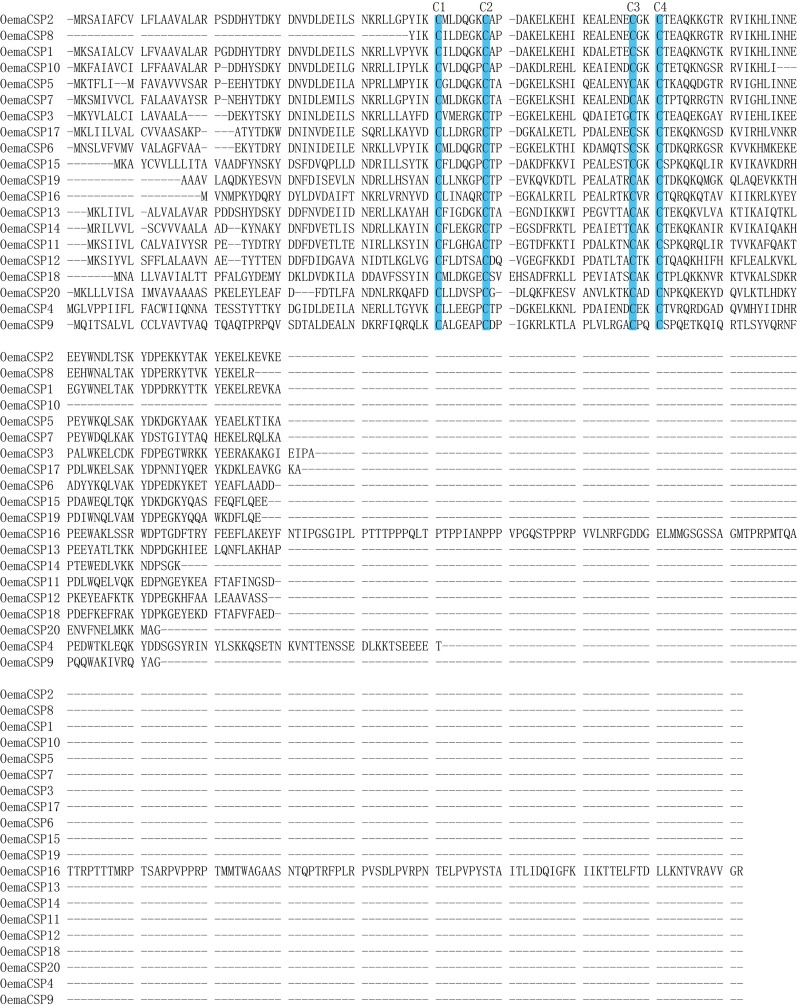
Aligned putative full ORF of CSP gene sequences of *O*. *emarginata*. Four conserved cysteines are highlighted in blue.

A total of 20 candidate chemosensory protein (CSP) genes were identified in *O*. *emarginata*, with a mean length of 128 aa. The full ORF of the 16 CSP genes were obtained (Table 3, Fig 5). In the phylogenetic tree, *OemaCSP9* and *OemaCSP16* were clustered the homologous genes of other insect species into two conserved groups (Fig 5). The bootstrap values of 5 CSPs (*OemaCSP1*, *2*, *7*, *8*, and *10*) were < smaller than 50%, although these were clustered with studied CSPs of the Lepidopteran species. Four conserved cysteines were found in all CSP genes, but *OemaCSP16* differed from the other CSPs in terms of the number of amino acids (Fig 6).

**Table 3:** BLASTp results of candidate chemosensory proteins of *O*. *emarginata*.

Gene name	Full ORF	FPKM value	ORF length (aa)	Reference gene ID	Reference gene name	E_value	Similarity (%)
*OemaCSP1*	Yes	3112	128	ABM67689.1	Chemosensory protein CSP2 [*S*. *exigua*]	1.43E-71	81.3
*OemaCSP2*	Yes	859	128	ABM67689.1	Chemosensory protein CSP2 [*S*. *exigua*]	2.46E-71	79.7
*OemaCSP3*	Yes	4257	127	ABB91378.1	Chemosensory protein [*H*. *assulta*]	2.33E-66	77.2
*OemaCSP4*	Yes	1278	150	AGY49270.1	Chemosensory protein [*S*. *inferens*]	1.49E-60	61.3
*OemaCSP5*	Yes	3729	125	AGH20053.1	Chemosensory protein 15 [*H*. *armigera*]	9.21E-58	81.6
*OemaCSP6*	Yes	415	123	AGR39578.1	Chemosensory protein 8 [*A*. *ipsilon*]	9.71E-69	79.7
*OemaCSP7*	Yes	324	127	AGY49267.1	Chemosensory protein [*S*. *inferens*]	4.81E-56	62.2
*OemaCSP8*	No	42	78	ABM67689.1	Chemosensory protein CSP2 [*S*. *exigua*]	5.81E-42	87.2
*OemaCSP9*	Yes	11	111	AGR39575.1	Chemosensory protein 5 [*A*. *ipsilon*]	4.94E-60	87.4
*OemaCSP10*	No	1	94	AAF71290.2	Chemosensory protein [*Mamestra brassicae*]	9.30E-45	71.3
*OemaCSP11*	Yes	1770	123	AIW65100.1	Chemosensory protein [*H*. *armigera*]	3.66E-64	71.5
*OemaCSP12*	Yes	13	122	BAF34359.1	Chemosensory protein 7 [*B*. *mori*]	7.07E-47	68.0
*OemaCSP13*	Yes	71	125	BAF34357.1	Chemosensory protein precursor [*B*. *mori*]	8.31E-44	69.6
*OemaCSP14*	No	4	109	AFR92094.1	Chemosensory protein 10 [*H*. *armigera*]	8.47E-64	90.8
*OemaCSP15*	Yes	904	120	AEX07267.1	CSP6 [*H*. *armigera*]	8.22E-64	81.7
*OemaCSP16*	Yes	19	293	AIW65104.1	Chemosensory protein [*H*. *armigera*]	5.67E-132	82.4
*OemaCSP17*	Yes	8	126	AIW65099.1	Chemosensory protein [*H*. *armigera*]	2.50E-73	87.3
*OemaCSP18*	Yes	106	122	BAG71920.1	Chemosensory protein 12 [*Papilio xuthus*]	1.31E-35	73.0
*OemaCSP19*	No	23171	110	AEX07265.1	CSP2 [*H*. *armigera*]	2.32E-65	87.3
*OemaCSP20*	Yes	485	107	AEX07268.1	CSP7 [*H*. *armigera*]	2.83E-30	52.3

**Fig 7:**
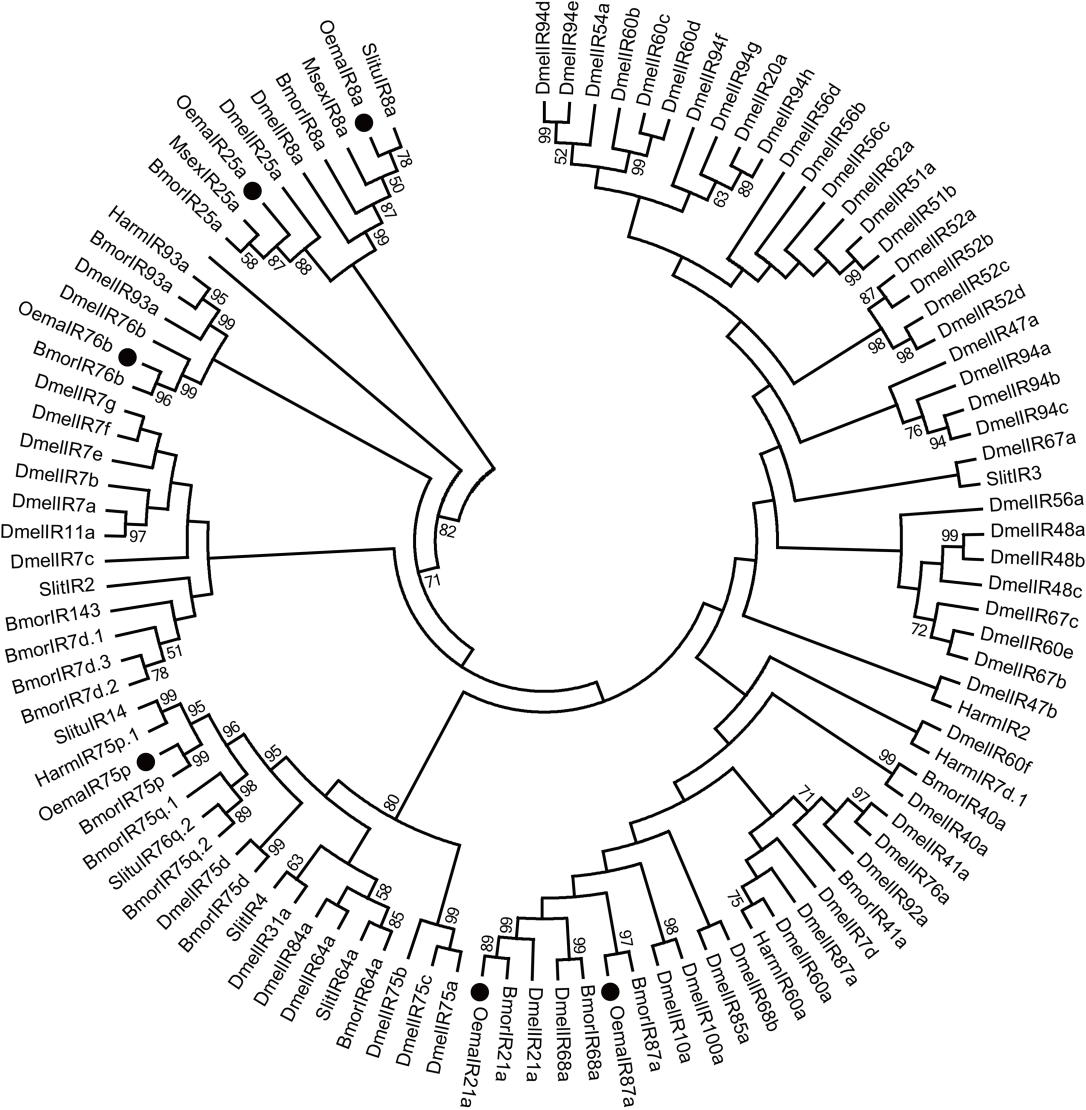
Phylogenetic analysis of putative IR gene sequences of *O*. *emarginata* (black circles). The tree is rooted with IR25a and IR8a lineages. Bootstrap values < 50% are not shown. Bmor, *B*. *mori*, Dmel, *D*. *melanogaster*, Harm, *H*. *armigera*, Msex, *M*. *sexta*, Oema, *O*. *emarginata*, Slitu, *S*. *litura*.

**Fig 8:**
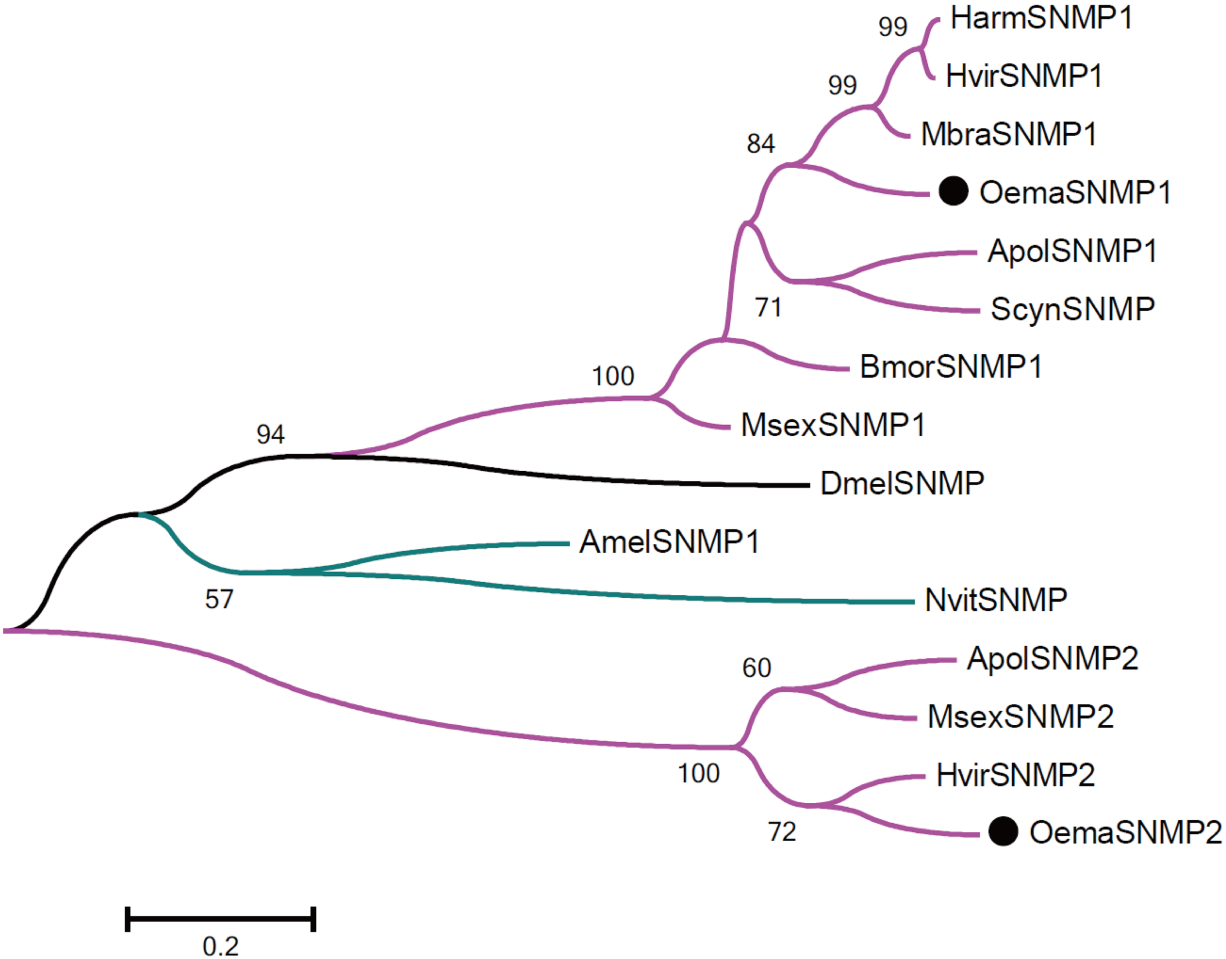
Phylogenetic analysis of putative SNMP gene sequences of *O*. *emarginata* (black circles), *D*. *melanogaster* (black lines), other moth species (purple lines), and Hymenopteran species (green lines). Bootstrap values < 50% are not shown. Amel, *Apis mellifera*, Apol, *Antheraea polyphemus*, Bmor, *B*. *mori*, Dmel, *D*. *melanogaster*, Harm, *H*. *armigera*, Hvir, *H*. *virescens*, Mbra, *M*. *brassicae*, Msex, *M*. *sexta*, Nvit, *Nasonia vitripennis*, Oema, *O*. *emarginata*, Scyn, *Samia ricini*, Slitu, *S*. *litura*.

Six candidate ionotropic receptor (IR) genes and 2 sensory neuron membrane protein (SNMP) genes were identified in *O*. *emarginata*, and their mean lengths were 535 aa and 522 aa, respectively (Tables 4 and 5). All *O*. *emarginata* IRs and SNMPs were clustered with Lepidopteran IRs and SNMPs, respectively, with the bootstrap values > 80% (Figs 7 and 8). The full ORF of 2 SNMP genes was obtained.

**Table 4:** BLASTp results of candidate ionotropic receptors of *O*. *emarginata*.

Gene name	Full ORF	FPKM	ORF length (aa)	Reference Gene ID	Reference gene name	E_value	Similarity (%)
*OemaIR21a*	No	15.8	514	ADR64678.1	Chemosensory ionotropic receptor IR21a [*S*. *littoralis*]	5.06E-180	51.9
*OemaIR25a*	No	9.5	910	AJD81628.1	Ionotropic receptor 25a, partial [*H*. *assulta*]	0	95.7
*OemaIR75p*	No	17.5	534	ADR64684.1	Chemosensory ionotropic receptor IR75p [*S*. *littoralis*]	6.11E-145	40.6
*OemaIR76b*	No	6.2	557	AGY49253.1	Putative ionotropic receptor [*S*. *inferens*]	0	73.8
*OemaIR87a*	No	4.6	277	ADR64689.1	Chemosensory ionotropic receptor IR87a [*S*. *littoralis*]	3.03E-125	69.0
*OemaIR8a*	No	14.8	575	AFC91764.1	Putative ionotropic receptor IR8a, partial [*Cydia pomonella*]	0	87.5

**Table 5:** BLASTp results of candidate SNMP genes of *O*. *emarginata*.

Gene name	Full ORF	FPKM	ORF length (aa)	Reference gene ID	Reference gene name	E_value	Similarity (%)
*OemaSNMP1*	Yes	19	525	AF462067_1	Sensory neuron membrane protein [*H*. *armigera*]	0	79.0
*OemaSNMP2*	Yes	505	518	AGN48099	Sensory neuron membrane protein 2 [*S*. *litura*]	0	73.0

### Expression of olfactory genes with RNA sequences

The FPKM values of the chemosensory receptors were < 60, and *OemaORco* showed the highest FPKM value (Tables 1 and 4). The FPKM value of *OemaOR29* was higher, but those of the other candidate PRs were lower than the general ORs, including *OemaOR14*, *25*, *27*, and *32* (Table 1). The FPKM values of *OemaIR75p* and *OemaIR21a* were larger than those of the co-receptors *OemaIR25a* and *OemaIR8a* (Table 4). In contrast to chemosensory receptors, 39.0% of the OBP and 52.4% of the CSP genes showed FPKM values > 300, including 3 candidate PBPs (Tables 2 and 3). *OemaPBP1* showed the highest FPKM value among all OBPs, and *OemaCSP19* had the highest FPKM value among all chemosensory genes. The FPKM value of *OemaSNMP1* was < 20, but that of *OemaSNMP2* was > 500 (Table 5).

### Expression of all olfactory genes between male and female antennae

**Fig 9:**
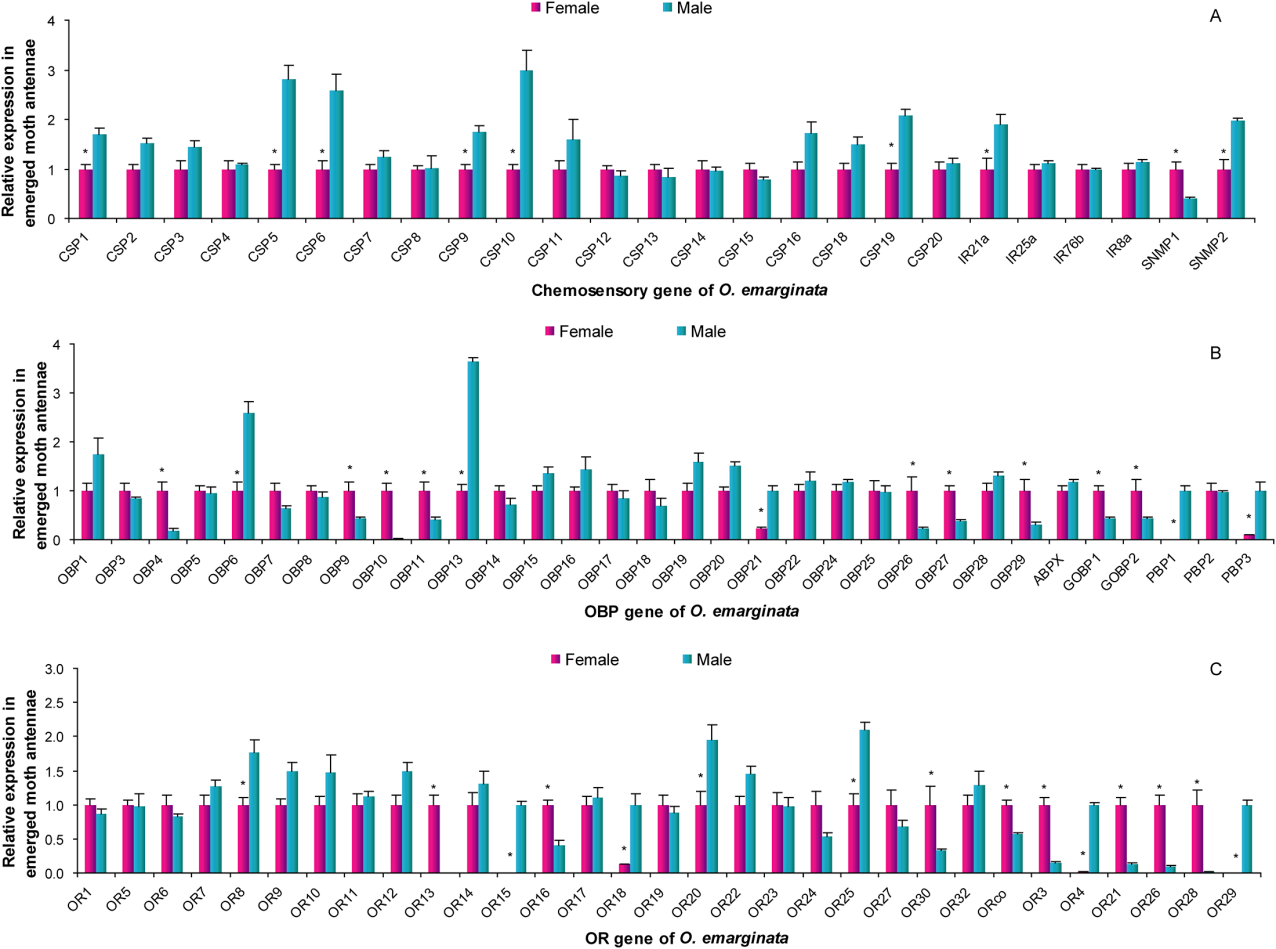
Expression levels of olfactory genes in male and female antennae as measured by RT-qPCR analysis. Gene expression was calculated relative to the reference genes, *UCCR* and *AK*. The expression in female antennae was arbitrarily defined as 1 for all genes and was used in the normalization of gene expression of the male antennae. A, Expression levels of *CSP*, *IR*, and *SNMP* genes. B, Expression levels of the *OBP* genes. C, Expression levels of *OR* genes.

Five candidate PRs (*OemaOR3*, *21*, *26*, *28*, and *30*), *OemaOR13*, *OemaOR16*, *OemaOR30*, *OemaORco*, 2 GOBPs, 7 OBPs (*OemaOBP4*, *9–11*, *26*, *27*, and *29*), and *OemaSNMP1* were expressed at significantly higher levels in females, and *OemaOR26*, *OemaOR28*, *OemaOR13*, and *OemaOBP10* were specifically expressed in females (Fig 9). Two candidate PRs (*OemaOR29* and *4*), *OemaOR18*, 4 general ORs (*OemaOR8*, *15*, *20*, and *25*), 2 PBPs (*OemaPBP1* and *3*), 3 OBPs (*OemaOBP6*, *13*, and *21*), 6 CSPs (*OemaCSP1*, *5*, *6*, *9*, *10*, and *19*), *OemaIR21a*, and *OemaSNMP2* were expressed at significantly higher levels in males compared to that in females, and *OemaOR29*, *OemaOR4*, *OemaOR18*, *OemaOR15*, *OemaPBP1*, and *OemaPBP3* were specifically expressed in males (Fig 9).

### Phylogeny of pheromone recognition genes of types I and II pheromones

**Fig 10:**
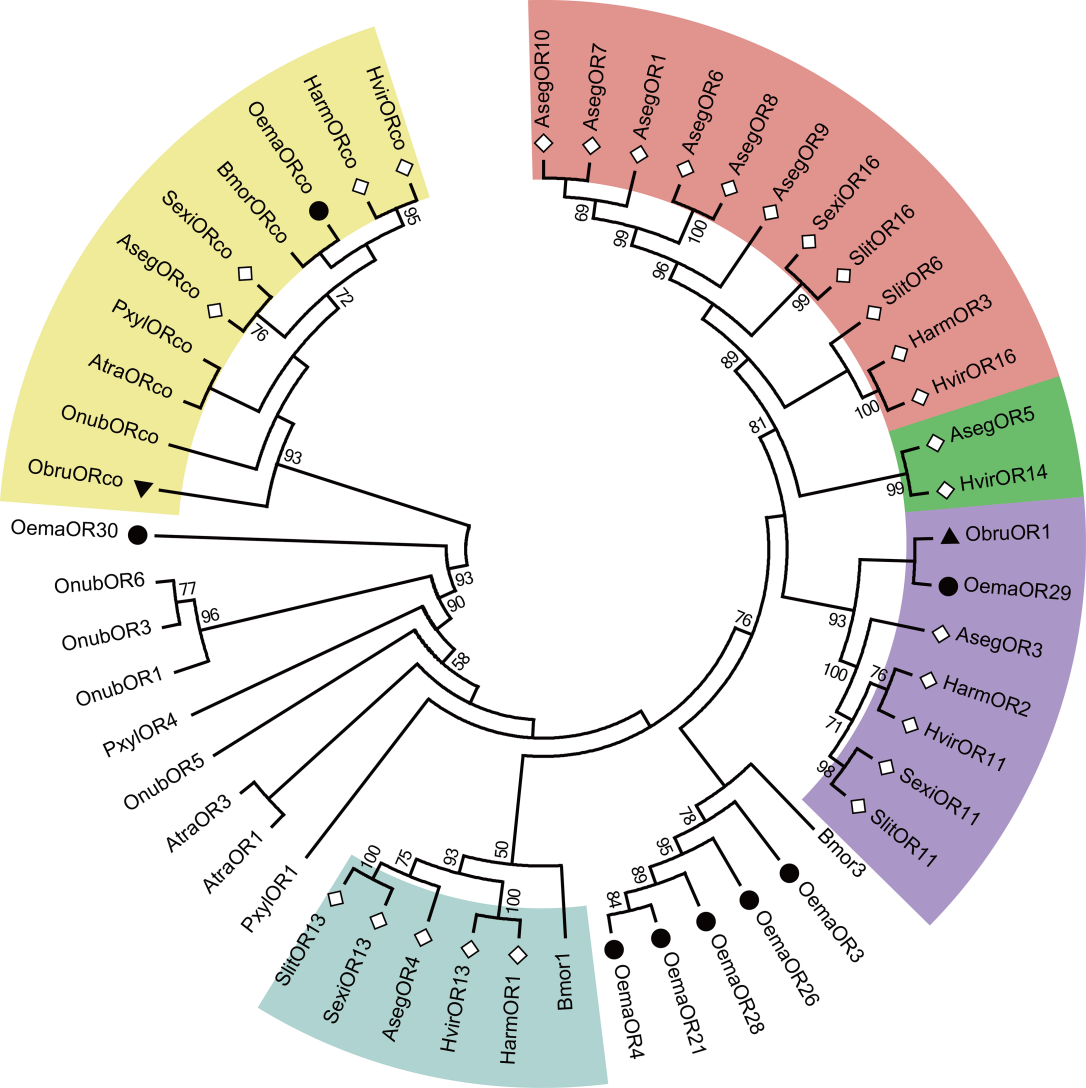
The phylogeny of Lepidopteran PRs. The tree was rooted with Orco lineage (yellow color). Bootstrap values < 50% are not shown. Genes of *O*. *emarginata*, *O*. *brumata*, and other noctuid species are indicated by black circles, black triangles, and diamonds, respectively. Clusters PRI—PRIV for type I pheromones are indicated in red, green, purple, and blue, respectively. Aseg, *A*. *segetum*, Atra, *Amyelois transitella*, Bmor, *B*. *mori*, Harm, *H*. *armigera*, Hvir, *H*. *virescens*, Obru, *O*. *brumata*, Oema, *O*. *emarginata*, Onub, *O*. *nubilalis*, Pxyl, *P*. *xylostella*, Sexi, *S*. *exigua*, Slit, *S*. *litura*.

In the phylogenetic tree, 4 orthologous PRs clusters for type I pheromones were obtained (Cluster PRI-PRIV), and candidate PRs of the noctuid species (excluding *O*. *emarginata*) formed subclusters of these 4 clusters, with high bootstrap support (≥ 89, Fig 10). *OemaOR29* and *ObruOR1* (the only identified pheromone receptor for type II sex pheromones from the geometrid *O*. *brumata*) belonged to cluster PRIII (Fig 10). Other candidate PRs of *O*. *emarginata* were not grouped with any of these 4 clusters, but 5 (*OemaOR3*, *4*, *21*, *26*, and *28*) were clustered, with a bootstrap support of 78 (Fig 10).

**Fig 11:**
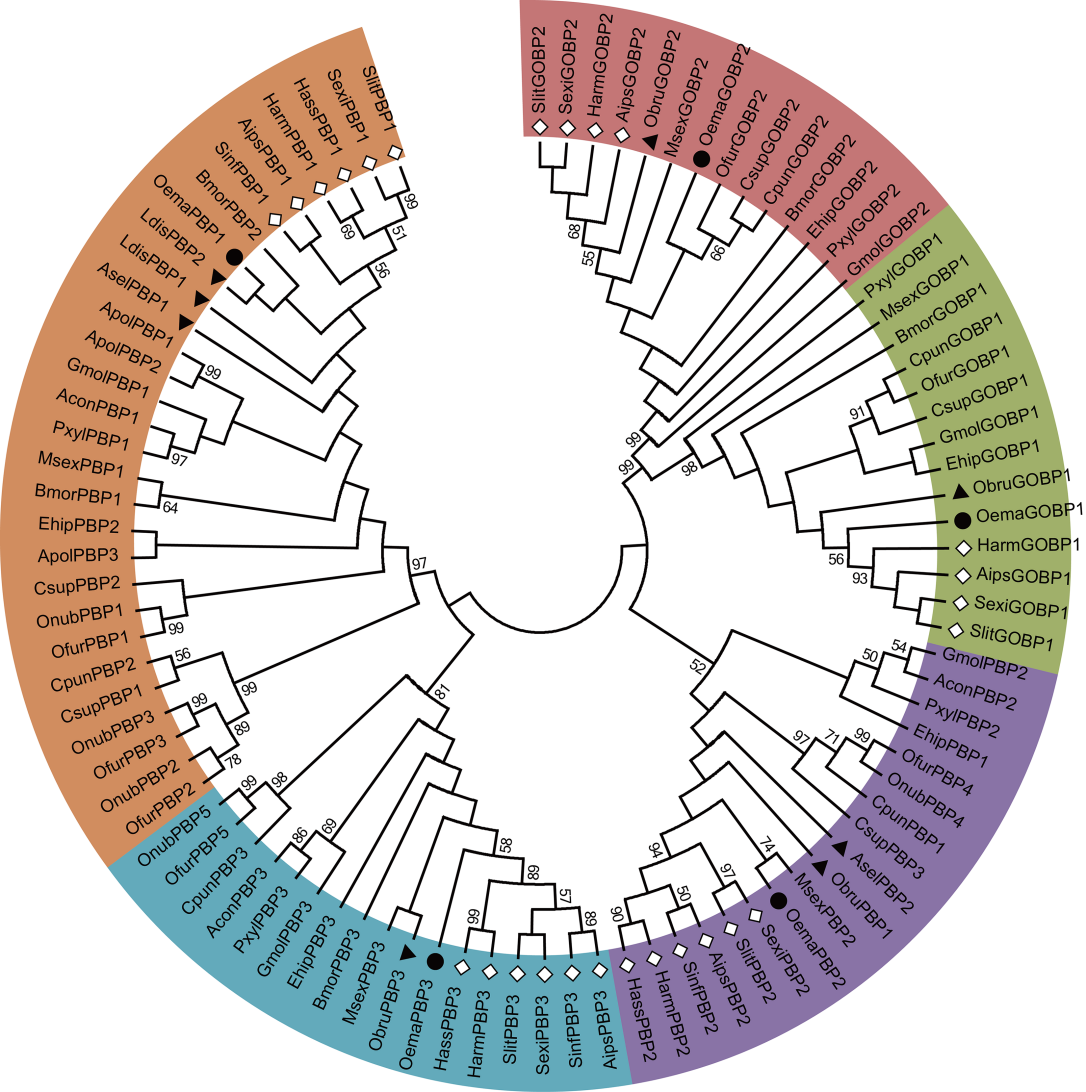
The phylogeny of Lepidopteran PBPs. The tree was rooted with GOBP lineage. Bootstrap values < 50% are not shown. Genes of *O*. *emarginata*, other species with type II pheromones, and the other noctuid species are indicated by black circles, black triangles, and diamonds, respectively. Clusters PBPI—PBPIII are indicated by orange, purple, and blue colors, respectively. Acon, *Argyresthia conjugella*, Aips, *A*. *ipsilon*, Apol, *A*. *polyphemus*, Asel, *Ascotis selenaria cretacea*, Bmor, *B*. *mori*, Cpun, *C*. *punctiferalis*, Csup, *C*. *suppressalis*, Ehip, *Eogystia hippophaecolus*, Harm, *H*. *armigera*, Hass, *H*. *assulta*, Gmol, *G*. *molesta*, Ldis, *Lymantria dispar*, Msex, *M*. *sexta*, Obru, *O*. *brumata*, Oema, *O*. *emarginata*, Ofur, *O*. *furnacalis*, Onub, *O*. *nubilalis*, Pxyl, *P*. *xylostella*, Sexi, *S*. *exigua*, Sinf, *S*. *inferens*, Slit, *S*. *litura*.

The PBPs and GOBPs of all test species were clustered into 3 (Cluster PBPI-PBPIII) and 2 (Cluster GOBPI-II) apparent clusters, with good bootstrap support (≥ 52), respectively (Fig 11). *OemaPBP3* and *OemaGOBP1* were clustered with orthologous PBPs and GOBP1s of the other noctuids for type I pheromones, respectively (bootstrap support ≥ 56) (Fig 11). However, *OemaPBP1*, *OemaPBP2*, and *OemaGOBP2* were not clustered within PBPs and GOBP2s from other noctuid species for type I pheromones. *OemaPBP2* was clustered with *MsexPBP2*, with a bootstrap value of 74 (Fig 11).

## Discussion

### The unique life history of *O*. *emarginata* might have driven the increase in the number of chemosensory genes

*O*. *emarginata* has a unique life history. The larvae feed on Menispermaceae plants, but adults suck on the juices of ripe fruits. Mating behavior is mediated by female sex pheromones. Mated females oviposit on Menispermaceae plants. Odorant classes from different species might thus be different [52]. Moths of *O*. *emarginata* must recognize a range of different odors with diverse chemical structures emitted from conspecifics, fruits, or orchard background and larval host plants. The olfactory acuity and discriminatory power in *O*. *emarginata* may have evolved to fulfill its ecological needs. We found 104 candidate olfactory genes in the antennae of *O*. *emarginata*, including 35 ORs, 41 OBPs, 20 CSPs, 6 IRs, and 2 SNMPs. In these 104 olfactory genes, 2 ORs (*OemaOR24* and *35*) and 5 CSPs (*OemaCSP1*, *2*, *7*, *8*, and *10*) were not effectively clustered with those of other Lepidopterans (bootstrap values < 50) in the phylogenetic analysis. In addition, 8 OemaORs (*OemaOR11*, *14*, *17*, *19*, *20*, *25*, *27*, and *32*) were clustered into the clade of *OfurOR34*, *MsexOR42*, and *AdisOR9* (bootstrap value = 87) (Fig 2), and 7 OemaOBPs (*OemaOBP4*, *11*, *13*, *18*, *23*, *27*, and *35*) were clustered with *AipsOBP4*, *SlitABP1*, *SlitOBP12*, *SexiABP1*, *HvirABP2*, *HarmOBP7*, and *HarmOBP7*.*2* (bootstrap value = 61) in the phylogenetic trees (Fig 3). Some of those genes might be species-specific to *O*. *emarginata* and used to recognize the odors produced by the Menispermaceae and fruits.

The number of chemosensory binding proteins (including OBPs and CSPs) was slightly smaller than in *B*. *mori*, which included the whole genome, but larger than in other moth species studied using the same protocol (antennal transcriptome). These other species included polyphagous insects such as *S*. *litura* (Table 6). The larger number of chemosensory binding proteins might be due to the life history of *O*. *emarginata* and the larger database in our study. We found a total of 103,301,292 reads that were assembled into 2,202,660 contigs, and compared to 55,288,304 reads assembled into 105,971 contigs in *S*. *litura* [51]. However, the number of chemosensory receptors was lower than in most other moths (Table 6). The low expression level of chemosensory receptor genes (FPKM < 60) and short read length (250 bp) of the transcriptome analysis might have resulted in short sequences for many chemosensory receptor genes. However, the long sequence of the chemosensory receptor genes (about 400 aa and 800 aa for OR and IR, respectively) [53,54] and the criterion of 50% ORF length cutoff might have excluded numerous chemosensory receptors with short sequences. No gustatory receptor gene was identified in the antennae, which suggests that the antennae of *O*. *emarginata* are not major taste organs. The proboscis, which harbors considerably fewer sensilla than antennae, are believed to specialize in taste reception in some moths [37,55]. In addition, the long sequence of gustatory receptor genes (about 400 aa) and the criterion of 50% ORF length cutoff might have excluded some gustatory receptors with short sequences.

**Table 6:** Chemosensory genes in insects.

Species	GR	OR	IR	OBP	CSP	SNMP	Reference
*A*. *ipsilon*	1	42	24	33	12	2	[25]
*B*. *mori*	65	66	18	46	22	1	[40,41]
*C*. *suppressalis*	/	47	20	26	21	2	[42]
*C*. *pomonella*	20	58	21	/	/	/	[43,44]
*D*. *houi*	/	33	10	23	17	2	[45]
*D*. *kikuchii*	/	33	9	27	17	2	[45]
*H*. *armigera*	/	60	19	34	18	2	[46]
*H*. *assulta*	/	64	19	29	17	2	[46]
*M*. *sexta*	1	47	6	18	19	2	[20]
*O*. *furnacalis*	5	56	21	23	10	2	[47,48]
*O*. *emarginata*	0	35	6	41	20	2	The study
*S*. *inferens*	/	39	3	24	24	2	[49]
*S*. *littoralis*	6	47	17	36	21	/	[50]
*S*. *litura*	/	26	9	21	18	/	[51]

/ means the number of genes in the family was not reported.

### Olfactory genes with sex-specific expression

We identified 2 candidate PRs (*OemaOR29* and *4*) and 2 candidate PBPs (*OemaPBP1* and *3*) that showed male-biased expression and might be involved with female sex pheromone recognition in *O*. *emarginata*. Our results were consistent with the study on the sex pheromone recognition in a sibling speciesm *O*. *excavate*, which produces two sex pheromone compounds at the ratio of 86:14[30]. *OemaOR29* was clustered with *ObruOR1* and *AsegOR3* in the phylogenetic tree, which recognized the pheromonal tetraene of *O*. *brumata*, 3Z,6Z,9Z-19:H and the triene 3Z,6Z,9Z-21:H separately [56]. *OemaPBP1* and *OemaPBP3* were ranked in the clusters PBPI and PBPIII in the phylogenetic analysis, respectively, which showed an equally consistent association with male-specific pheromone sensitive sensilla [57]. Orthologous genes in the clusters PBPI and PBPIII play critical and minor roles in female sex pheromone perception, respectively [58–61]. *OemaOR29* and *OemaPBP1* showed the highest FPKM values in all ORs and OBPs, respectively, and might be used to recognize the main sex pheromone component. *OemaOR4* and *OemaPBP3* might be involved in the recognition of the minor sex pheromone component. Further studies are needed to verify the function of these genes.

Five candidate pheromone receptor genes (*OemaOR3*, *21*, *26*, *28*, and *30*) showed female-biased expression, and *OemaOR26*, and *OemaOR28* were specifically expressed in females. The function of these genes is unknown, but these might be used by females to recognize male pheromones. Production of short-range pheromones has been reported in male butterflies [62]; these function in female mate selection, act as an aphrodisiac, and arrest female departure [63,64].

Besides the candidate PR genes, some genes with sex-specific expression were detected; for example, *OemaOR13* was female-specific. These genes might also be correlated with sex specific behaviors such as the recognition of oviposition cues by females [65–67].

### Diversification of olfactory recognition to sex pheromones

Type II pheromones have mainly been found in the moth superfamilies Geometroidea and Noctuoidea [17], but olfactory genes for type II pheromones were only identified in the geometrids *A*. *selenaria cretacea* [68,69] and *O*. *brumata* [56] and the erebids *L*. *dispar* [70–72] and *Hyphantria cunea* [73]. The sex pheromone of female *O*. *emarginata* was not published, but it was similar to the epoxide components of a preliminary identification (Du et al., unpublished data). In addition, cis-9,10-epoxy-(Z)-6 -heneicosene and cis-9,10-epoxy-(Z, Z)-3,6-heneicosadiene were identified as the major and minor sex pheromone components from a sibling species, *O*. *excavate* [30]. In the present study, 7 candidate PRs and 3 candidate PBPs were obtained from the noctuid *O*. *emarginata* using antennal transcriptome analysis.

The diversification of olfactory recognition to sex pheromones has been verified for type I pheromones in noctuids such as *A*. *segetum*, *H*. *armigera*, and *S*. *litura*, and the phylogeny of moth PRs and PBPs for type I pheromone identified several apparent orthologous clusters (cluster PRI—PRIV for PRs and cluster PBPI—PBPIII for PBPs). PRs and PBPs from different clusters specifically respond to different type I sex pheromone components [59,74]. Although the functions of PRs for type II pheromone recognition were not identified, phylogenetic analysis clustered 3 candidate PRs of *H*. *cunea* [73] and 7 candidate PRs of *O*. *emarginata* into three groups. These findings are indicative of the diversification in olfactory recognition to type II pheromones.

Phylogenetic analysis did not separate the PRs and PBPs for types I and II pheromones, thereby suggesting that PRs and PBPs for types I and II pheromones evolved from a common ancestor. However, type I pheromones differed from type II pheromones in its chemical characteristics. *OemaOR29* and *ObruOR1* belonged to cluster PRIII of type I pheromone recognition, which is under strong purifying selection (a very small dN/dS values), and did not respond to any type I sex pheromone components [75]. On the contrary, *ObruOR1* was verified to specifically recognize the pheromonal tetraene of *O*. *brumata*, 3Z,6Z,9Z-19:H, and the orthologous receptor *AsegOR3* responded strongly to the triene 3Z,6Z,9Z-21:H instead of any female sex pheromone of *A*. *segetum* [56]. Cluster III might be specialized in the recognition type II sex pheromone components. In addition, 6 other candidate PRs of *O*. *emarginata* were not grouped within any of the four PR clusters of type I sex pheromones, but 5 of these were grouped into a specific cluster, with a bootstrap support value of 78. The candidate main sex pheromone-binding protein *OemaPBP1* was not clustered into the subgroup of PBP1 genes from other noctuid species in the phylogenetic tree. These results indicate that the olfactory genes for sex pheromones in *O*. *emarginata* might differ from those of other noctuid species, and the diversification of pheromone recognition genes for types I and II sex pheromones might exist in noctuid species.

## Conclusions

A total of 104 candidate olfactory genes, including 7 candidate PRs and 3 candidate PBPs were identified from the noctuid *O*. *emarginata*. Seven olfactory genes of *O*. *emarginata* were not effectively clustered with those of other Lepidoptera, and OemaORs and OemaOBPs in 2 clusters were strongly expanded. These changes in olfactory genes in *O*. *emarginata* might correlate with its unique life history. Most candidate PRs and PBPs (except for *OemaOR29* and *OemaPBP3*) of *O*. *emarginata* were not clustered with other noctuid species. *OemaOR29* was grouped into cluster PRIII of type I pheromones, which recognized type II pheromones instead of type I pheromones. Noctuid species might thus have undergone diversification of the pheromone recognition gene for types I and II sex pheromones. Our results increase our understanding of the molecular mechanism of *O*. *emarginata* olfaction and the evolution of olfactory genes associated with sex pheromones.

## Supporting information

S1 Fig GO annotation. (TIF)

S1 Table Primers used in this study. (DOC)

S1 File Amino acid sequences of the olfactory genes used in the phylogenetic analysis. (TXT)
