# Supplementary figures and images for: Antennal transcriptome analysis of the piercing moth Oraesia emarginata (Lepidoptera: Noctuidae)
Source: PLoS One. 2017 Jun 14;12(6):e0179433. doi: 10.1371/journal.pone.0179433 (PMC5470721; doi:10.1371/journal.pone.0179433)

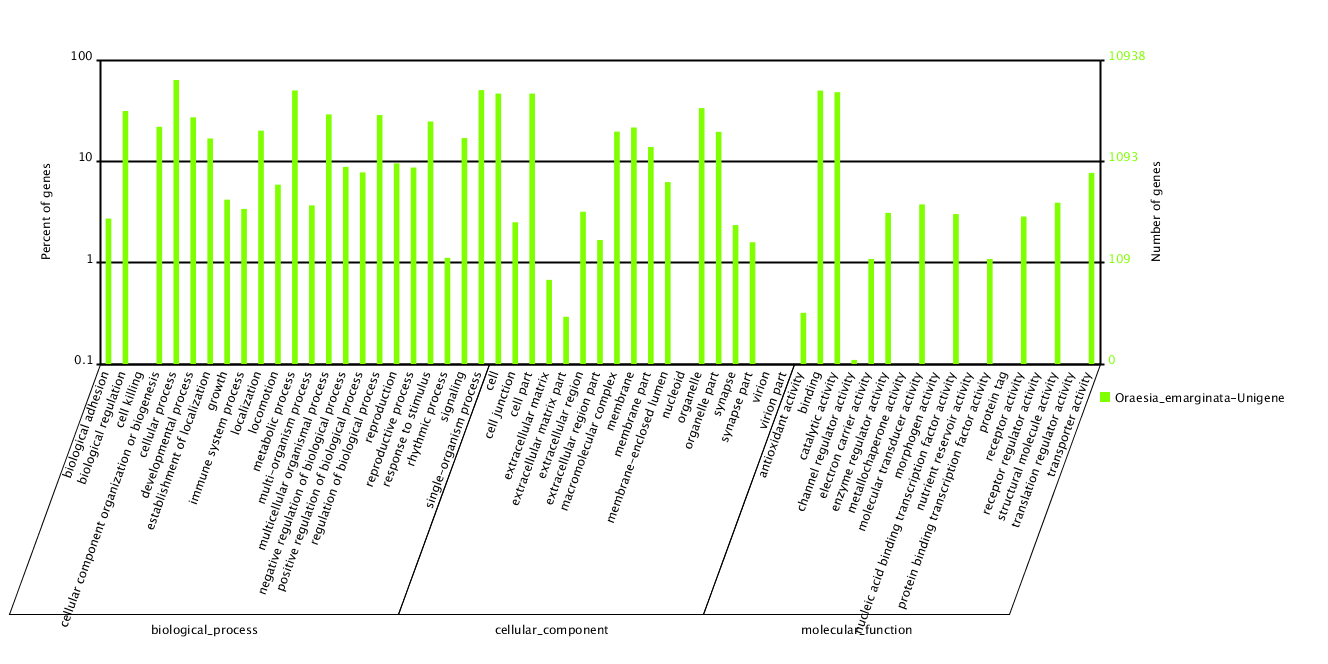

Supplement: S1 Fig — (TIF) [file pone.0179433.s001.tif]
