# Supplementary material for: Antennal transcriptome analysis of the piercing moth Oraesia emarginata (Lepidoptera: Noctuidae)
Source: PLoS One. 2017 Jun 14;12(6):e0179433. doi: 10.1371/journal.pone.0179433 (PMC5470721; doi:10.1371/journal.pone.0179433)
Supplement: S1 Table — (DOC) [file pone.0179433.s002.doc]

S1 Table. Primers used in the study

| Gene name | Forward primer (5'-3') | Reverse primer (5'-3') | Tm (^o^C) | Efficiency (%) | R² |
| --- | --- | --- | --- | --- | --- |
| *OemaAK* | CGACGACCACTTCCTGTTCA | GCATGGAGATGAGACGGAGG | 60 | 99.29 | 0.993 |
| *OemaABPX* | TGTATCGGGACTCCCAGTGT | AGCAAGCTCTTCTGCCTTCT | 60 | 99.89 | 0.989 |
| *OemaCSP1* | TGATCAACAACGAGGAGGGC | GTGGTGTACTTCCTGTCGGG | 60 | 99.25 | 0.994 |
| *OemaCSP10* | CATTTGCCGCAGTCGTTTTC | TACCTCAAGTGTGTGCTCGAC | 60 | 90.97 | 0.976 |
| *OemaCSP11* | AAGACAAACTGCGCCAAGTG | TGTGAAAGCCTCCTTGTACTCG | 60 | 90.77 | 0.999 |
| *OemaCSP12* | ATGCTACACTGACAGCATGC | GCGGCGAAATGTTTTCCTTC | 60 | 91.24 | 0.993 |
| *OemaCSP13* | ACGACGACTTCAACGTTGAC | TGTTTCTCCGTGCATTTGGC | 60 | 92.21 | 0.994 |
| *OemaCSP14* | TCACAAGGTCTTCCCATTCCG | ACCACTTGCGCCAAATGTAC | 60 | 91.14 | 0.994 |
| *OemaCSP15* | TGCACGCCTGATGCTAAAGA | TCCCGCACGTTGATTCAAGA | 60 | 92.58 | 0.995 |
| *OemaCSP16* | CTACTAGCGCGAGACCTGTG | CGACCGACTACTGCTCGTAC | 60 | 85.44 | 0.977 |
| *OemaCSP18* | ACAAAGGCGAATGCAGTGTG | TGGCGCATGATGTTGCAATC | 60 | 92.96 | 0.974 |
| *OemaCSP19* | AGCAAATGGGCAAGCAGTTG | CCTTCCACGCCTGTTGGTAT | 60 | 98.57 | 0.988 |
| *OemaCSP2* | ATGCTCGACCAAGGAAAGTG | TCCGTACATTTGCCGCATTC | 60 | 93.31 | 0.996 |
| *OemaCSP20* | GCTATCATGGTAGCCGTTGC | CGAAGTCGAATGCCTCCAGA | 60 | 89.39 | 0.980 |
| *OemaCSP3* | AAGTACGTTCTAGCGCTGTG | TTTCAGCTCCTTGCCTTCAG | 60 | 94.64 | 0.985 |
| *OemaCSP4* | AACAACGCCACAGAGTCATC | TAGACGTTCATTCGCGAGGATC | 60 | 95.07 | 0.994 |
| *OemaCSP5* | ACGGCAAGGAGCTTAAATCG | TGGTTTTGAGTTCGGCTTCG | 60 | 95.10 | 0.993 |
| *OemaCSP6* | TGGTGCCGTACATTAAGTGC | AAGTTTGCATGGCGTCCTTG | 60 | 97.91 | 0.986 |
| *OemaCSP7* | TCATCTCCAGGTCGATGTTGTC | ATCGTTGTCTGTCTGTTCGC | 60 | 84.55 | 0.974 |
| *OemaCSP8* | TCCAGTGCTCCTCCTCATGA | AACGAGTGTGGCAAATGCAC | 60 | 81.15 | 0.989 |
| *OemaCSP9* | ATCGGCAAGCGATTGAAGAC | TGTGGGAAGTTTCTCTGCAC | 60 | 97.98 | 0.989 |
| *OemaGOBP1* | TGAGCAAGCACTACAACCTG | TGTTCTTTTGCACGCACGTC | 60 | 99.96 | 0.976 |
| *OemaGOBP2* | TTTTGGGAAGGCGCTTGAAG | TTGGACATGCAGATGATGGC | 60 | 86.14 | 0.987 |
| *OemaIR21a* | GACGATGCTGCTTTGGAATCTC | ATTTGCCATGGGTCCAAGAG | 60 | 70.26 | 0.985 |
| *OemaIR25a* | CGGATGCCAAAGCCTTTTTG | TGCCTCAAATCACCGACTTG | 60 | 97.96 | 0.989 |
| *OemaIR76b* | TAAAGGCGTGGCTTTCGTTG | TTCAGCATCATGACCCACAC | 60 | 63.74 | 0.985 |
| *OemaIR8a* | TCTGGTTTGCACTCACATCG | AACTGCTCCAAGGATGAGACTG | 60 | 88.80 | 0.995 |
| *OemaOBP1* | TGTTTCGTGGCTGTGACCTT | GCCAACCTCCTTAGCCGATT | 60 | 95.17 | 0.996 |
| *OemaOBP10* | AACGCTCCTTGCTTTTTGGC | TTTTGCAGAATGCCGGCATC | 60 | 69.25 | 0.997 |
| *OemaOBP11* | AGAACGACGCAGACATGGAG | ATCCCAGCTCTTTCACAGCC | 60 | 63.23 | 0.982 |
| *OemaOBP13* | TTGCTACCTGTGAAGCGATG | TTTTGGCTGGCACGATTTCC | 60 | 92.97 | 0.975 |
| *OemaOBP14* | TGCTGTATCCGGCTGACTTG | ACATTTCGCTGTCCAGTACGA | 60 | 98.32 | 0.985 |
| *OemaOBP15* | ATGAAAGCGCCTGTCAAAGC | ACGCCTCGCAGATATCTTTG | 60 | 85.76 | 0.972 |
| *OemaOBP16* | TCACGGAAGATCAAGTCGGC | TCCGTCACACATGTCGTTGT | 60 | 98.73 | 0.993 |
| *OemaOBP17* | AAGAATCCCGCGGGCAAATA | CTCCCATTCGGTCTCGACTG | 60 | 99.35 | 0.991 |
| *OemaOBP18* | CGATTGTGCCAAAGTGAACG | ACGCCAAAGTTGAGTGCTTC | 60 | 99.08 | 0.991 |
| *OemaOBP19* | GCACAACTTCGACCTCAAAGAC | TGGCAAGAACCGCTTTGATG | 60 | 92.56 | 0.996 |
| *OemaOBP20* | ACTTTGGACGTCATCAGCAG | TGCTGGCTTGTTAGGTGTTG | 60 | 92.27 | 0.997 |
| *OemaOBP21* | TTTTGCCTGATGGAGGAAGC | TGTCTTTGTCCGGGGTATCAAG | 60 | 87.49 | 0.987 |
| *OemaOBP22* | TGACAAGGCAACAGCTGAAG | AACTTGCCCTGCTCGATTTG | 60 | 88.78 | 0.988 |
| *OemaOBP24* | ACAAACGCTAAGGGTGTTGC | TCCCAGTTGTTGCTGACTTG | 60 | 86.74 | 0.983 |
| *OemaOBP25* | CCGTTCACTCACGACGAGAA | AGCCGACAAGACCTTCCAAG | 60 | 98.81 | 0.995 |
| *OemaOBP26* | AGTTGAATAGAGCGGTGTACGG | TTGAACATCGACGCGAACTC | 60 | 65.64 | 0.931 |
| *OemaOBP27* | ACGGTGTTTTGTTTGGGAAGC | CTGACCCGGCTTCAAGAAGT | 60 | 90.74 | 0.998 |
| *OemaOBP28* | TTGCTCGTGTTTGCTGCTTG | AGCGCGTACTTCTTCAGTTG | 60 | 98.66 | 0.988 |
| *OemaOBP29* | GTATTGGAGAGCTGTGCCGA | AGGGCTTTCTTGTCGTAGCC | 60 | 64.82 | 0.936 |
| *OemaOBP3* | TTGTGACTGTGGCTAATGCG | TGCACTGAATGTCGTTGCAC | 60 | 98.59 | 0.978 |
| *OemaOBP4* | CGGAATGCACTGAGAAACTTGG | TCAACGCCGTCACTAACATC | 60 | 68.35 | 0.981 |
| *OemaOBP5* | TGGTGAACGCAACGGTAAAC | ACATGTAGGCTCTCTCACATGG | 60 | 90.41 | 0.988 |
| *OemaOBP6* | AGGAGCGTTTATTGCCTGAC | TCGCATGAGTCCACACATTC | 60 | 95.20 | 0.994 |
| *OemaOBP7* | CTAGCCTGCGCTTACAAAGC | TGGCAAGTCTATTCTCGTCACC | 60 | 97.95 | 0.984 |
| *OemaOBP8* | CCGGGATGATGAACGACCAA | AGTCAGGAGCCTTTTCCACG | 60 | 83.11 | 0.989 |
| *OemaOBP9* | AATTCACCGCCGAATGCATC | AAGAACCTTCTTCGCAGCAG | 60 | 79.97 | 0.962 |
| *OemaOR1* | AACGACAGGATCTTGGGAGAAG | TGATGAGCGTATTCGGGGAA | 60 | 86.31 | 0.987 |
| *OemaOR10* | TGTTTGTGCTGTACCTCACC | TGGCGATCACGATCATGATG | 60 | 95.09 | 0.993 |
| *OemaOR11* | TATCCGCTTGCTGCAATCTC | ATGAAAGTGGCCAGCAATCC | 60 | 71.80 | 0.990 |
| *OemaOR12* | CGTGGAGCAAGAAAATTGGC | TATCGGCTACCAGGCTGCTA | 60 | 83.26 | 0.994 |
| *OemaOR13* | TTCGGAGACCACTAACCTGAAC | ATCAGAAACGGCAGCATCAG | 60 | 76.70 | 0.978 |
| *OemaOR14* | AACTGAGTCGTGATGTGCGT | AATTGTGGTGTGGTGGAACG | 60 | 90.69 | 0.996 |
| *OemaOR15* | TATGAGGTTCTCGAGGCCTTTG | AGTAAACAGCTAGCCGCAAC | 60 | 93.33 | 0.997 |
| *OemaOR17* | AACAACGCTCTACTGATCGC | ATCGAAAGGCATCCAGTACTCG | 60 | 90.84 | 0.967 |
| *OemaOR18* | TGTACGCGGGCATTTTGATG | TTGGCACGAGAACAATGCAC | 60 | 95.08 | 0.996 |
| *OemaOR19* | AACGAATGGGCATACAAGGC | AAATCGCGCTGTAACTGTCC | 60 | 86.37 | 0.991 |
| *OemaOR20* | TCTGCCCTGCTTAGGAGTCT | GATGAGATGCTCTCCCTCGC | 60 | 81.50 | 0.999 |
| *OemaOR21* | TCGATGAAGACGCACAAACG | TCACGTTGAAGGCACTGTTG | 60 | 95.06 | 0.995 |
| *OemaOR22* | TGAGAGGCCCAAATGCTGTATG | CACGGCTCTTTCAAACTGTACG | 60 | 93.80 | 0.989 |
| *OemaOR23* | TTTCGCACTTCGTGATGGTC | TGCGAGCACAGAGTACTGAC | 60 | 80.20 | 0.993 |
| *OemaOR24* | TGTCACGCAAGCATCTCTGT | TCACTATCCATGTGCGCCAG | 60 | 67.93 | 0.974 |
| *OemaOR25* | TAACGCACATCACCATGCAG | TGCTTGGCACGAGAGTTTAC | 60 | 91.27 | 0.995 |
| *OemaOR26* | AATAGCGCTTTAGCCCTGAC | AAGCAAGTTCACGAGCACTG | 60 | 85.52 | 0.991 |
| *OemaOR27* | AGTGCACCGCATCATAAGTG | TTGCTGCCCAAGATTGCATC | 60 | 92.23 | 0.998 |
| *OemaOR28* | ACCTTACCACCTTTCCACTTCC | TGGCCACTTCTATCGATTCGTC | 60 | 83.36 | 0.992 |
| *OemaOR29* | TTCAACTTAACGCCCGTGAC | AACACAAACGCTGCTTGCAC | 60 | 85.59 | 0.979 |
| *OemaOR3* | CAGAAGCATTTGGACCGATGT | GCATGATGACGGTGATTGGC | 60 | 69.84 | 0.989 |
| *OemaOR30* | TACGCATTCAGTTCCGTCTG | TGCCCTGATTTCCTGTGTACTC | 60 | 90.81 | 0.968 |
| *OemaOR32* | TTGTTCCAGAAGTCGCTTGC | TCCTGTTCGAATGCATGCTC | 60 | 90.92 | 0.974 |
| *OemaOR4* | TGTCTCCACATTCGAGTTGGG | ACCTCTGATGATTGCCACGG | 60 | 80.30 | 0.995 |
| *OemaOR5* | AGCGCTTTCAGTTTGGCTTC | AAATGCCCATCCACACCATC | 60 | 83.80 | 0.999 |
| *OemaOR6* | TGCATCGGCGTATTTAGGAC | TCGTGGCGAATTAGACAACG | 60 | 90.64 | 0.997 |
| *OemaOR7* | CCGCCGCTCAAGATTGTTTC | ATCGTCTCCAGCAGCTCAAC | 60 | 81.01 | 0.999 |
| *OemaOR8* | AATACGACACCGCAACGATG | TACGACACCGCAACGATGAA | 60 | 86.34 | 0.999 |
| *OemaOR9* | TGTGGATGGCACCTCACTTC | GCTGTTGAGCTCGCAGGATA | 60 | 90.12 | 0.995 |
| *OemaORco* | ATCGGCCGAATACTGCTGAG | GCTCCTCTAAGCGTCATCCC | 60 | 98.50 | 0.990 |
| *OemaPBP1* | TTGGAAGGAGGGTTTCGAGC | ATCAGCTGCTTCGCCATCTT | 60 | 93.79 | 0.988 |
| *OemaPBP2* | TCAAGTGCACCATGGAGACG | AAGTCCGCCTTGTTAGCGTT | 60 | 89.88 | 0.980 |
| *OemaPBP3* | GCTATGAAACATGGCGCTGG | ACTCGTCTTCGTTGGAGCTG | 60 | 94.67 | 0.987 |
| *OemaSNMP1* | TCGTTATGCCGCATGTGTTC | TTGAGTGCAGACAGCTTTCG | 60 | 89.54 | 0.980 |
| *OemaSNMP2* | TGCCTTGCTAACAGTTCACG | TGCCGTCAAACAACAGATCC | 60 | 99.25 | 0.994 |
| *OemaUCCR* | TACCCTGACTTCTCGGCGTA | CTCGGCCAGCTTGATCTCAA | 60 | 99.35 | 0.996 |
